# Persistent Inflammatory Stimulation Drives the Conversion of MSCs to Inflammatory CAFs That Promote Pro-Metastatic Characteristics in Breast Cancer Cells

**DOI:** 10.3390/cancers13061472

**Published:** 2021-03-23

**Authors:** Linor Rubinstein-Achiasaf, Dina Morein, Hagar Ben-Yaakov, Yulia Liubomirski, Tsipi Meshel, Eti Elbaz, Orly Dorot, Edward Pichinuk, Michael Gershovits, Miguel Weil, Adit Ben-Baruch

**Affiliations:** 1 The Shmunis School of Biomedicine and Cancer Research, George S. Wise Faculty of Life Sciences, Tel Aviv University, Tel Aviv 6997801, Israel; linoru@gmail.com (L.R.-A.); Dinamorein1@gmail.com (D.M.); etzion.hagar1@gmail.com (H.B.-Y.); yulialiub@gmail.com (Y.L.); tsipi.meshel@gmail.com (T.M.); elbaztetro@gmail.com (E.E.); weilmiguel@gmail.com (M.W.); 2 Blavatnik Center for Drug Discovery, Tel Aviv University, Tel Aviv 6997801, Israel; orly.dorot@gmail.com (O.D.); eddypichinuk@gmail.com (E.P.); 3 The Nancy and Stephen Grand Israel National Center for Personalized Medicine, Weizmann Institute, Rehovot 7610001, Israel; gershovm@weizmann.ac.il

**Keywords:** breast cancer, cancer-associated fibroblasts, inflammation, interleukin 1β, mesenchymal stem cells, tumor necrosis factor α

## Abstract

**Simple Summary:**

Breast cancer progression is prominently regulated by the persistent presence of inflammatory mediators and by stromal cells that are located in the tumors. Here, we connected these two elements by demonstrating that potent pro-inflammatory cytokines (tumor necrosis factor α and interleukin 1β) lead to the conversion of mesenchymal stem cells (MSCs) to inflammatory cancer-associated fibroblasts (CAFs). These inflammation-driven CAFs secrete metastasis-promoting factors that elevate the dispersion, scattering, and migration of breast cancer cells via activation of tumor cell receptors that signal through Ras proteins and via Gαi proteins; the latter receptors were identified as the chemokine receptors CCR2, CCR5, and CXCR1/2. Together, these findings demonstrate that, in breast tumors, chronic inflammation can induce the deleterious process of MSC-to-CAF conversion and thus sets pro-inflammatory mediators as key targets for inhibition, potentially leading to lower levels of pro-metastatic CAFs in breast tumors.

**Abstract:**

The pro-inflammatory cytokines tumor necrosis factor α (TNFα) and interleukin 1β (IL-1β) are expressed simultaneously and have tumor-promoting roles in breast cancer. In parallel, mesenchymal stem cells (MSCs) undergo conversion at the tumor site to cancer-associated fibroblasts (CAFs), which are generally connected to enhanced tumor progression. Here, we determined the impact of consistent inflammatory stimulation on stromal cell plasticity. MSCs that were persistently stimulated by TNFα + IL-1β (generally 14–18 days) gained a CAF-like morphology, accompanied by prominent changes in gene expression, including in stroma/fibroblast-related genes. These CAF-like cells expressed elevated levels of vimentin and fibroblast activation protein (FAP) and demonstrated significantly increased abilities to contract collagen gels. Moreover, they gained the phenotype of inflammatory CAFs, as indicated by the reduced expression of α smooth muscle actin (αSMA), increased proliferation, and elevated expression of inflammatory genes and proteins, primarily inflammatory chemokines. These inflammatory CAFs released factors that enhanced tumor cell dispersion, scattering, and migration; the inflammatory CAF-derived factors elevated cancer cell migration by stimulating the chemokine receptors CCR2, CCR5, and CXCR1/2 and Ras-activating receptors, expressed by the cancer cells. Together, these novel findings demonstrate that chronic inflammation can induce MSC-to-CAF conversion, leading to the generation of tumor-promoting inflammatory CAFs.

## 1. Introduction

Luminal-A breast cancers (BC) are identified by the expression of hormone receptors (estrogen receptors (ER) and progesterone receptors (PR)) and by the absence of HER2 expression; although this disease subtype is considered as having a relatively favorable outcome, a considerable number of patients experience local recurrence and metastasis [1,2,3]. Similar to many other cancer types, the progression of luminal-A tumors is driven by many elements of the tumor microenvironment (TME) and by the way they interact with each other and with the cancer cells [4,5,6]. At the TME, major roles are attributed to chronic inflammation [7,8,9,10] and to stromal cells [11,12,13,14,15] in promoting disease progression in many cancer diseases, including luminal-A BC. However, so far, there is only a limited knowledge on the way these elements interact and affect pro-metastatic activities in this subtype of the disease.

Two pro-inflammatory cytokines have been recently strongly connected to poor disease outcome in BC: tumor necrosis factor α (TNFα) [16,17,18] and interleukin 1β (IL-1β) [19,20,21]. Our past research and studies by other investigators demonstrated that the two cytokines were minimally expressed in healthy breast tissues but that they were present in the majority of breast tumors, many of which were of the luminal-A subtype; moreover, in patient samples, TNFα and IL-1β were often simultaneously expressed from the stage of ductal carcinoma in situ and onwards, and their expression was significantly associated with advanced disease course [22,23,24,25,26,27]. A large number of studies indicate that TNFα and IL-1β promote disease progression by acting directly on the tumor cells and by affecting different components of the TME, including immune cells [16,17,18,19,20,21]. Together, the different observations propose that the persistent presence of TNFα and IL-1β at the tumor site contributes to chronic inflammation in BC and that the two cytokines play key roles in advancing disease course. 

These findings motivated us to determine the impact of continuous stimulation by TNFα + IL-1β on stromal cells. In this respect, we turned our attention to mesenchymal stem cells (MSCs). MSCs were demonstrated in remote metastases of BC patients [28] and were found to be derived from different sources in tumors. Among others, bone marrow (BM)-derived MSCs are known to be recruited to the tumor area, where they exert many tumor-promoting activities and undergo conversion to cancer-associated fibroblasts (CAFs) [12,14,29,30,31,32,33]; in general, these activated fibroblasts were reported as being heavily engaged in pro-metastatic tasks [11,33,34,35]. Recent studies provided evidence of the existence of heterogeneous CAF populations that can reside side by side in tumors, expressing different markers and functions [36,37,38,39]. 

The biology and origins of CAFs have been the subject of intensive research; however, much is still unknown regarding processes of MSC-to-CAF conversion. TNFα and IL-1β, known to be expressed by cancer cells and inflammatory cells and to constitute a part of the TME, can increase the tumor-stimulating properties of MSCs (TNFα [40,41,42,43,44,45,46] and IL-1β [44,45,46,47,48]); however, the ability of these two cytokines to induce MSC-to-CAF conversion and the impact of the resulting CAFs on tumor progression have not been investigated in depth. Therefore, in this study, we asked if persistent stimulation by the pro-inflammatory cytokines TNFα + IL-1β regulates the conversion process of MSCs to CAFs; what the phenotype of the resulting CAFs is; and whether they express pro-metastatic characteristics that can promote the metastatic phenotype of luminal-A breast tumor cells. 

Here, we demonstrate that persistent stimulation of BM-derived MSCs by TNFα + IL-1β leads to their conversion to CAFs and acquires the characteristics of inflammatory CAFs. These inflammatory CAFs release factors that promote the scattering and migratory functions of luminal-A BC cells. Moreover, we find that factors released by the inflammatory CAFs elevate the migration of luminal-A cells through cooperativity between Ras-activating receptors and specific chemokine receptors that are expressed by the cancer cells. 

Together, these findings reveal key roles for chronic inflammation in regulating stromal cell plasticity and the conversion of MSCs to tumor-supporting inflammatory CAFs. Here, pro-inflammatory cytokines induce the process on their own, independent of any other factors that may reside at the TME. These findings emphasize the important contribution of inflammatory mediators to tumor progression and set them as putative targets in cancer therapy.

## 2. Results

### 2.1. Following Persistent Stimulation by TNFα + IL-1β, MSCs Acquire a CAF-Like Morphology Accompanied by Modifications in Fibroblast-Related Transcriptional Programs

**Figure 1:**
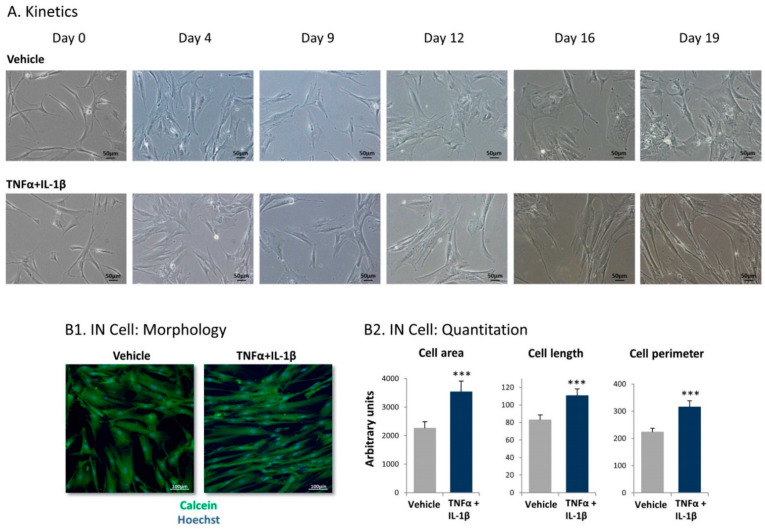
Following persistent stimulation of mesenchymal stem cells (MSCs) by tumor necrosis factor α (TNFα) + interleukin 1β (IL-1β), the resulting cells acquire elongated cancer-associated fibroblast (CAF)-like morphology. Human MSCs were exposed to TNFα (50 ng/mL) + IL-1β (0.5 ng/mL) or to vehicles. Cytokine concentrations were selected based on the considerations described in the Materials and Methods section. (**A**) At different time points, the cells were photographed by light microscopy. Images from a representative experiment out of *n* > 3 are presented. Bar, 50 μm. (**B**) Determination of cell characteristics using IN Cell technology in cells that were treated using the cytokines/vehicles for 18 days and were then subjected to IN Cell analysis. (**B1**) Cell morphology was detected by calcein (green) and Hoechst (blue) staining. Images of cell morphology from a representative experiment out of *n* = 3 are presented. Bar, 100 µm. (**B2**) Quantification of cell characteristics by the IN Cell technology. *** *p* < 0.001. The results of a representative experiment of *n* = 3 are presented.

In view of the continuous presence of TNFα and IL-1β together in many luminal-A breast tumors [23,24], we began this study by determining the impact of TNFα + IL-1β stimulation on the morphology of stimulated MSCs. Kinetics analyses that were performed until day 19 of cytokine/vehicle treatment provided evidence to a gradual process, leading to an elongated CAF-like morphology of the cells after persistent stimulation of the MSCs by the two cytokines (Figure 1A). From here on, we generally used persistent stimulation by TNFα + IL-1β for 14–18 days, with similar results within this time range. Of note, the elongated CAF-like morphology of the cells following TNFα + IL-1β stimulation was also clearly distinguished in fluorescent analyses (Figure 1(B1)). To enable quantitative analysis of the morphological changes, we used the IN Cell technology, indicating that, following continuous stimulation of the MSCs by TNFα + IL-1β, cell area, length, and perimeter were significantly increased (Figure 1(B2)). 

**Figure 2:**
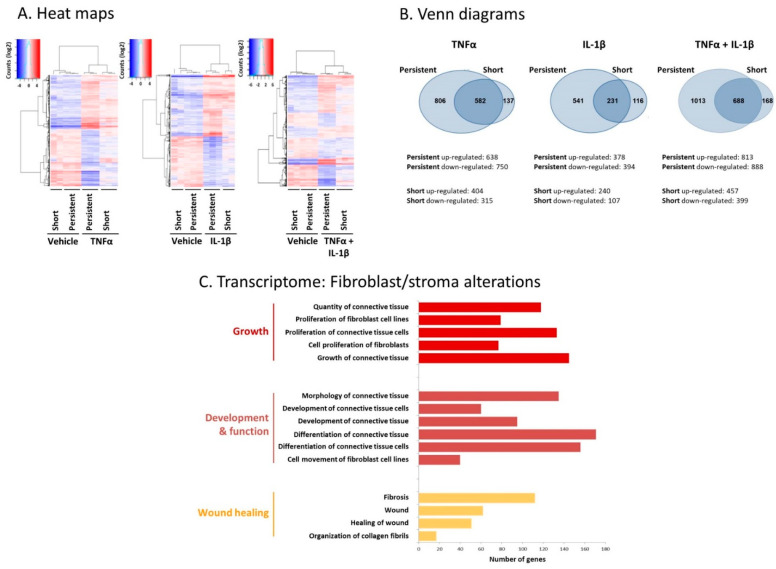
Persistent stimulation of MSCs by TNFα + IL-1β leads to prominent alterations in gene expression and in fibroblast-relevant transcriptional programs in the resulting CAF-like cells. Human MSCs were exposed to “persistent” stimulation by TNFα and/or IL-1β for 14 days or to “short” stimulation of 48 h (concentrations as in Figure 1) in three independent biological repeats, followed by RNAseq analyses. Control cells were treated using the vehicles of the cytokines. (**A**) Heat maps. Deseq-normalized counts, values were centered. (**B**) Venn diagrams. Upregulated genes: FC > 2, padj < 0.05; downregulated genes: FC < 0.5, padj < 0.05. (**C**) Fibroblast-relevant transcriptional programs that were significantly modified by persistent TNFα + IL-1β stimulation, at FC > 2 or FC < 0.5 and padj < 5 × 10^−10^, are demonstrated.

In parallel, transcriptome analyses were performed on MSCs that were persistently treated by TNFα + IL-1β or by vehicles for 14 days (Tables S1 and S2 demonstrate the top 60 upregulated genes and the top 60 downregulated genes, respectively). To more precisely pinpoint modifications in gene expression that represent processes resulting from continuous cytokine stimulation, transcriptome analyses were performed in parallel on MSCs that were stimulated by TNFα + IL-1β for short stimulation of 48 h only. Here, the gene expression analysis demonstrated prominent and most significant changes in the transcriptome of the CAF-like cells resulting from persistent TNFα + IL-1β stimulation compared to vehicle-treated cells. First, the heat maps in Figure 2A demonstrate that the alteration levels in gene expression were stronger in MSCs that were exposed to persistent TNFα + IL-1β stimulation compared to short stimulation. Then, as presented in Venn diagrams (Figure 2B), the persistent stimulation by TNFα + IL-1β gave rise to alterations in a higher number of genes than short-term exposure to the two cytokines: the transcription levels of a total of 1701 genes were modified by persistent stimulation by TNFα + IL-1β vs. 856 genes in total that were modified by short TNFα + IL-1β stimulation (Figure 2B). Of interest, the expression of 1013 genes was exclusively modified by continuous TNFα + IL-1β stimulation, whereas the expression of only 168 genes was altered exclusively upon short TNFα + IL-1β stimulation. 

Moreover, following short-term stimulation but even more prominently after continuous stimulation using the cytokines, the effects on gene expression were considerably stronger when the MSCs were stimulated by TNFα + IL-1β together compared to stimulation by each cytokine individually: whereas the transcription levels of 1701 genes in total were modified by persistent stimulation using both TNFα + IL-1β, transcription of a total of 1388 genes was altered by persistent TNFα stimulation alone and the transcription of 772 genes in total was modified after persistent IL-1β treatment alone (Figure 2B). Thus, the most pronounced alteration in gene expression was noted upon persistent stimulation of MSCs by TNFα + IL-1β together compared to all other conditions, supporting the concomitant and persistent use of TNFα + IL-1β together in all further analyses. These findings are in line with our preliminary studies that revealed the higher ability of the combined TNFα + IL-1β stimulation to induce a CAF-like morphology on continuously stimulated MSCs than each cytokine alone. 

Furthermore, the transcriptome analyses indicated that major alterations in stroma/fibroblast-related transcriptional programs were observed in the CAF-like cells generated following continuous stimulation of MSCs using TNFα + IL-1β (Figure 2C). Between others, changes were noted in the expression of genes involved in the regulation of fibroblast proliferation and movement as well as of fibrosis and development of connective tissue, which are fundamental processes related to fibroblast activities in cancer. 

### 2.2. Following Persistent Stimulation by TNFα + IL-1β, MSCs Undergo Conversion to Inflammatory CAFs

**Figure 3:**
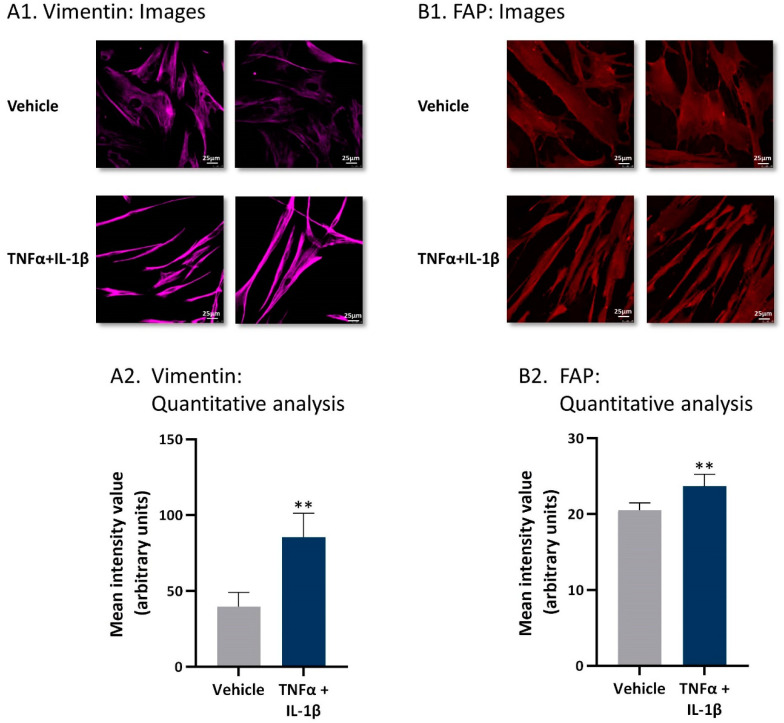
Following persistent stimulation of MSCs with TNFα + IL-1β, the resulting CAF-like cells express typical CAF markers. Human MSCs were exposed to persistent TNFα + IL-1β stimulation (concentrations as in Figure 1) or to vehicles, generally for 14–18 days. The expressions of vimentin (**A**) and fibroblast activation protein (FAP) (**B**) were determined by confocal analyses. (**A1**,**B1**) Two representative images are demonstrated for each treatment, derived from a representative experiment out of *n* = 3. Bar, 25 μm. (**A2**,**B2**) Quantitative analyses of the fluorescence intensity performed by the ImageJ program on images of the representative experiment (*n* ≥ 5 images for each treatment). ** *p* < 0.01.

To follow up on the above findings, we determined the impact of persistent stimulation by TNFα + IL-1β on the characteristics and functional properties of the resulting CAF-like cells. Here, we first determined the effects of continuous cytokine stimulation on the expression of typical CAF markers using the CAF-like cells. The images and quantitative analyses of confocal studies indicated that the expression levels of vimentin were elevated in MSCs that have been exposed to prolonged TNFα + IL-1β stimulation (Figure 3A). In parallel, the expression levels of fibroblast-activation protein (FAP) were also elevated upon continuous TNFα + IL-1β stimulation, as indicated by the images and quantitative analyses of confocal studies (Figure 3B). Together, the findings on vimentin and FAP indicate that MSCs that were stimulated consistently by TNFα + IL-1β acquired a CAF phenotype. Moreover, in line with findings demonstrating that CAFs that are derived from BM-MSCs expressed low levels of PDGFRa, COL1A1, and FSP-1 [49], the BM-MSCs used in our studies demonstrated higher levels of these three genes compared to the CAF-like cells obtained following continuous stimulation with TNFα + IL-1β (Figure S1). 

**Figure 4:**
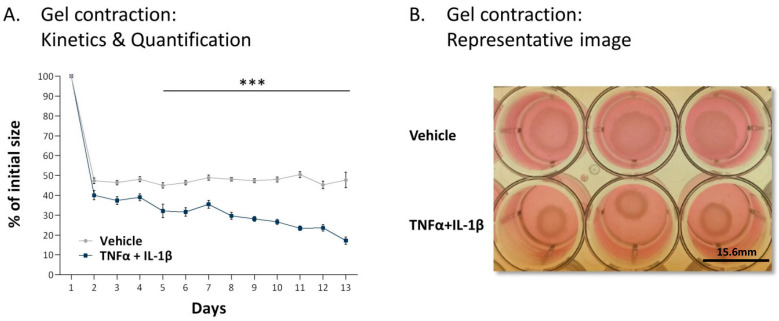
Following persistent stimulation of MSCs with TNFα + IL-1β, the resulting CAF-like cells express typical CAF functions. Human MSCs were exposed to persistent TNFα + IL-1β stimulation (concentrations as in Figure 1) or to vehicles, generally for 14–18 days. The figure demonstrates the results of a collagen contraction assay, in which the change in collagen gel size was determined along the process. (**A**) Kinetics analyses of changes in collagen gel diameter compared to original gel size, measured by ImageJ. The average ± SEM of 5 independent experiments is demonstrated. *** *p* < 0.001. (**B**) An image from a representative experiment out of *n* = 5 is presented. Three wells are demonstrated for each treatment. The photo was taken at day 10 of the collagen contraction assay. Bar, 15.6 mm.

These phenotypic and gene expression analyses strongly suggest that the persistent inflammatory stimulation leads to the conversion of MSCs to activated CAFs. This possibility has gained a definite support using collagen contractions assays (Figure 4). Here, we observed that the MSCs exposed to persistent TNFα + IL-1β stimulation acquired a most prominent ability to contract collagen gels, a most typical functional property of activated fibroblasts. Together, the above morphological and functional findings indicate that persistent stimulation by TNFα + IL-1β leads to the generation of CAFs through a MSC-to-CAF conversion process. 

**Figure 5:**
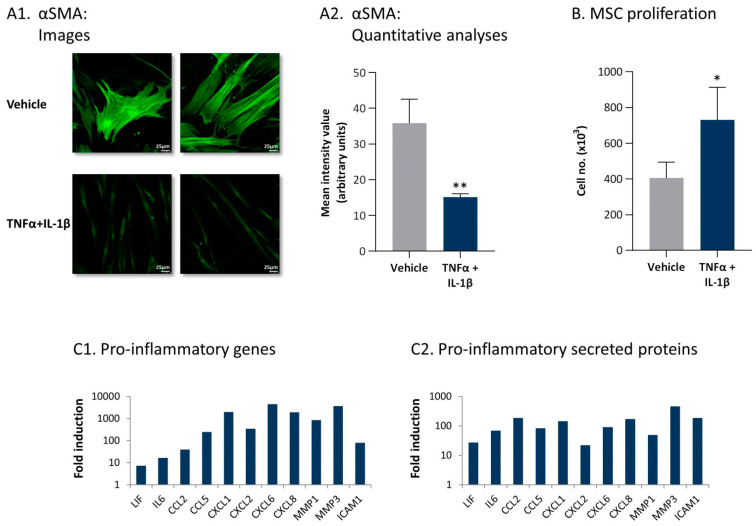
Persistent stimulation with TNFα + IL-1β leads to the conversion of MSCs to inflammatory CAFs. (**A**,**B**) Human MSCs were exposed to persistent TNFα + IL-1β stimulation (concentrations as in Figure 1) or to vehicles, generally for 14–18 days. (**A**) αSMA expression was determined by confocal analyses. (**A1**) Two images of each treatment, derived from a representative experiment out of *n* > 3, are presented. Bar, 25 μm. (**A2**) Quantitative analyses of the fluorescence intensity performed by the ImageJ program on images of the representative experiment (*n* ≥ 5 images for each treatment). ** *p* < 0.01. (**B**) Cell proliferation was determined by cell counts at the end of the stimulation process. Average ± SD of *n* > 3 is presented. * *p* < 0.05. Time-dependent analysis of MSC cell numbers along the stimulation process are demonstrated in Figure S2. (**C**) Expression of pro-inflammatory genes and secreted proteins, determined at the end of the stimulation process, is demonstrated. (**C1**) mRNA expression was determined by transcriptome analyses performed at day 14, with three independent biological repeats. padj < 10 × 10^−10^–padj < 5 × 10^−271^, depending on the gene. (**C2**) Expression of secreted proteins, determined by secretome analyses performed at days 18–19 with three independent biological repeats. *p* < 10^−3^–*p* < 7.5 × 10^−7^, depending on the protein. Data are presented as the fold induction of values obtained in cytokine-stimulated cells compared to vehicle-treated cells for all genes and proteins. Tables S1–S4 demonstrate the 60 top upregulated or downregulated genes and proteins, obtained following persistent stimulation of the MSCs by TNFα + IL-1β.

To follow up on our above findings demonstrating that the continuous TNFα + IL-1β stimulation leads to the generation of CAFs and in view of recent studies on CAF plasticity [36,37,38,39], we then asked what is the phenotype of the inflammation-driven CAFs. In line with the characteristics of inflammatory CAFs identified in pancreatic ductal adenocarcinoma (PDAC) and the major roles of IL-1 in their generation [50,51], we found out that the inflammation-driven CAFs expressed typical phenotypic characteristics of inflammatory CAFs. The images and quantitative analyses of Figure 5 demonstrate that, in comparison to MSCs exposed to vehicle, MSCs that were persistently stimulated by TNFα + IL-1β (1) expressed much lower levels of αSMA (Figure 5A); (2) had increased proliferation rates (Figure 5B), as demonstrated also in a time-dependent analysis (Figure S2); and (3) expressed elevated mRNA levels of inflammatory genes, including interleukin-6 (IL-6) and leukemia inhibitory factor (LIF) (Figure 5(C1); *p* values are given in Figure legend).

Specifically, data from transcriptome analyses indicated that CAFs that were derived from persistent stimulation of MSCs by TNFα + IL-1β expressed elevated levels of inflammatory and pro-metastatic chemokines including the CC chemokines CCL2 and CCL5 as well of the ELR + CXC chemokines CXCL1, CXCL2, CXCL6, and CXCL8 (Figure 5(C1)). In addition, high mRNA levels of matrix metalloproteinases (MMPs) and of the secreted form of ICAM-1—considered to be an indicator of pro-inflammatory conditions—were detected in MSCs that underwent persistent stimulation by TNFα + IL-1β (Figure 5(C1)).

**Figure 6:**
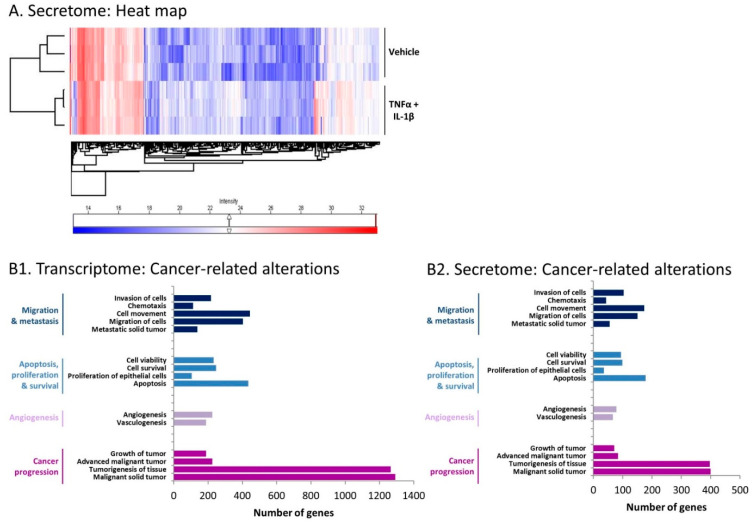
Following persistent stimulation of MSCs with TNFα + IL-1β, cancer-relevant programs are promoted in the resulting inflammatory CAFs. Human MSCs were exposed to persistent TNFα + IL-1β stimulation (concentrations as in Figure 1) or to vehicles for 14 days in transcriptome analyses and for 18–19 days in secretome analyses. (**A**) Heat maps of protein expression determined by secretome analysis of three independent biological repeats, comparing cytokine-stimulated cells and control cells. (**B**) Disorder analysis of cancer-related genes and secreted proteins for which expression was modified in cytokine-stimulated cells compared to vehicle-treated cells. (**B1**) Gene expression data were obtained by RNAseq analysis, in which genes were filtered using a cutoff of FC > 2 or FC < 0.5, padj < 5 × 10^−10^. (**B2**) Data of secreted proteins were obtained by secretome analysis, in which proteins were filtered using a cutoff of FC ≥ 2 or FC ≤ 0.5, *p* < 0.05. The INGENUITY program was used to identify affected disorders. Indicated disorders obeyed the cutoff of *p* < 10^−17^ in transcriptome analyses and *p* < 5 × 10^−8^ in secretome analyses.

Then, in view of the fact that factors secreted by the inflammation-driven CAF-like cells may impact the tumor cells or their TME, we performed secretome analyses. To this end, conditioned media (CM) that were devoid of exogenously added recombinant TNFα/IL-1β (termed herein “cytokine-devoid CM”) were obtained from the CAFs that were generated by persistent stimulation by TNFα + IL-1β and were subjected to secretome analyses in comparison to CM of control vehicle-exposed cells. These analyses demonstrated that the expression levels of many proteins were modified in the CM of cytokine-stimulated compared to vehicle-treated cells (Figure 6A). The secretome analyses indicated that the expression of 388 secreted proteins was significantly upregulated in CM of the inflammatory CAFs by fold change (FC) ≥ 2 (*p* < 0.05), whereas 79 proteins were significantly downregulated by FC ≤ 0.5 (*p* < 0.05) (Tables S3 and S4 demonstrate the top 60 upregulated secreted proteins and top 60 downregulated secreted proteins, respectively). Of importance was the fact that the secretome analyses (Figure 5(C2)) validated the results of the transcriptome studies (Figure 5(C1)), demonstrating that the same inflammatory elements that were upregulated in the inflammation-driven CAFs at the mRNA level were also prominently secreted by the cells, with high levels of IL-6, LIF, inflammatory chemokines, MMPs, and ICAM-1 noted (Figure 5(C2)). 

Together, the above findings provide evidence of an inflammation-driven process, in which MSCs that were stimulated consistently using inflammatory cytokines undergo conversion to CAFs that carry an inflammatory phenotype, and thus, they are characterized in our study as inflammatory CAFs.

### 2.3. Inflammation-Driven CAFs Express Prominent Alterations in the Expression of Cancer-Related Genes and Proteins

In view of the powerful roles of CAFs in regulating cancer progression [11,33,34,35], we explored the possibility that CAFs obtained following persistent stimulation of MSCs by TNFα + IL-1β undergo alterations in the expression of cancer-related genes. Indeed, as demonstrated in Figure 6(B1), the inflammation-driven CAFs demonstrated prominent changes in the expression levels of genes that control migration and invasion, apoptosis, proliferation and survival, angiogenesis, and tumor progression, where all indicated disorders obeyed the cutoff of *p* < 5 × 10^−17^. In parallel, secretome analysis of the inflammatory CAFs demonstrated pronounced modifications in the same pathways at the cutoff of *p* < 5 × 10^−8^ (Figure 6(B2)), with outstanding similarity to the gene expression pattern (Figure 6(B1)). These findings indicate that continuous stimulation of MSCs by TNFα + IL-1β greatly modified the expression of genes related to malignancy and of their corresponding secreted proteins. 

### 2.4. Inflammation-Driven CAFs Secrete Factors That Promote Pro-Metastatic Activities in Luminal-A BC Cells

We next determined the ability of CM derived from inflammatory CAFs to affect the tumor-promoting activities of luminal-A BC cells. To this end, at the end of the persistent growth period of MSCs with the cytokines, the resulting CAFs were grown for an additional 48 h in cytokine-devoid medium, and their CM were then applied to the tumor cells in parallel to CM of control vehicle-exposed cells. 

**Figure 7:**
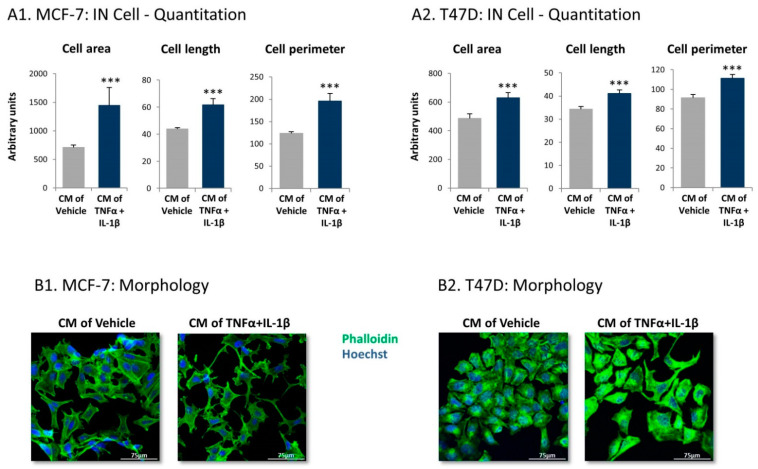
Following persistent stimulation of MSCs with TNFα + IL-1β, the resulting inflammatory CAFs release factors that induce morphological changes in luminal-A breast cancers (BC) cells. Human MSCs were exposed to persistent TNFα + IL-1β stimulation (concentrations as in Figure 1) or to vehicles, generally for 14–18 days. At the end of the stimulation period, cytokines were replaced by fresh cytokine-devoid media, and 48 h later, conditioned media (CM) were collected and administered to human luminal-A BC cells. (**A**) Tumor cell characteristics were quantified 4 days or 3 days after the addition of CM to MCF-7 cells (**A1**) and to T47D cells, repectively (**A2**) by IN Cell technology. The results of a representative experiment out of *n* = 3 are presented. *** *p* < 0.001. (**B**) Tumor cell morphology was photographed by confocal microscopy 2–3 days after the addition of CM, using phalloidin staining of actin filaments (green) and Hoechst staining of nuclei (blue). (**B1**) MCF-7 cells. (**B2**) T47D cells. Images from a representative experiment out of *n* = 3 are presented. Bar, 75 μm.

Using IN Cell technology, we first determined if MCF-7 and T47D human luminal-A BC cells undergo morphological changes upon growth with factors released by the inflammation-driven CAFs. Indeed, the quantitative IN Cell analyses demonstrated that, following growth with CM of the inflammatory CAFs, the tumor cells underwent significant modifications in cell area, length, and perimeter, all being increased (Figure 7A). The changes in cell morphology were evidenced also by confocal analyses, which demonstrated pronounced alterations in cell organization (Figure 7B). Upon growth with cytokine-devoid CM of inflammation-driven CAFs, the cancer cells exhibited definite cellular protrusions, acquired a dispersed organization and dissociated from each other (Figure 7B). 

**Figure 8:**
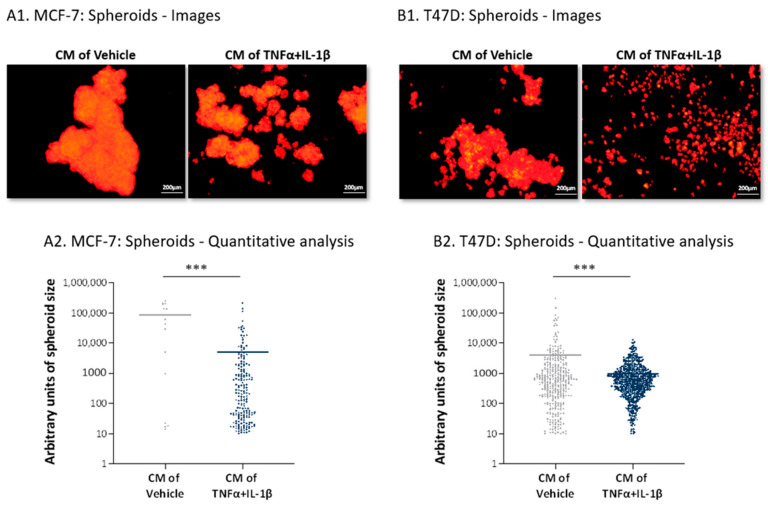
Following persistent stimulation of MSCs with TNFα + IL-1β, the resulting inflammatory CAFs release factors that promote scattering of luminal-A BC cells. Human MSCs were exposed to persistent TNFα + IL-1β stimulation (concentrations as in Figure 1) or to vehicles, generally for 14–18 days. Cytokine-devoid CM were collected as described in Figure 7 and were administered to mCherry-expressing MCF-7 (**A**) and T47D (**B**) human luminal-A BC cells, followed by determination of tumor cell scattering out of spheroids (MCF-7 cells) or the ability to form spheroids (T47D cells). (**A1**,**B1**) Representative images of spheroid formation at days 8 or 4 for MCF-7 cells and T47D cells are demonstrated, respectively. Bar, 200 µm. The results of a representative experiment out of *n* = 3 are presented. (**A2**,**B2**) Quantitative analyses of spheroid sizes performed by the ImageJ program on images of the representative experiment (*n* ≥ 7 images for each treatment). *** *p* < 0.001.

The dispersed distribution of the cancer cells following their growth with CM of inflammatory CAFs was also evidenced in the study of tumor spheroids. In this test, the tumor cells were grown in conditions that did not allow them to adhere to substrates; thus, they tended to create large spheroids by forming cell-to-cell contacts (similar to the approach taken in [52]). Using this test, we determined the ability of the cancer cells to spread out from spheroids (MCF-7 cells) or to form the spheroids (T47D cells) when incubated with cytokine-devoid CM of the inflammatory CAFs. The spheroid images of the cancer cells in Figure 8(A1,B1) and the quantification of their sizes (Figure 8(A2,B2)) clearly demonstrate that, whereas cancer cells grown with the CM of control MSCs formed large solid spheroids in which tight cell-to-cell contacts were formed, tumor cells that were treated by inflammatory CAF-derived cytokine-devoid CM exhibited dispersion abilities (MCF-7 cells; Figure 8A) or remained in dispersed organization characterized by small cell aggregates (T47D cells; Figure 8B). This type of arrangement of the inflammatory CAF-CM-treated cancer cells may attest to a preference towards elevated scattering, which may serve their needs well when they metastasize. 

**Figure 9:**
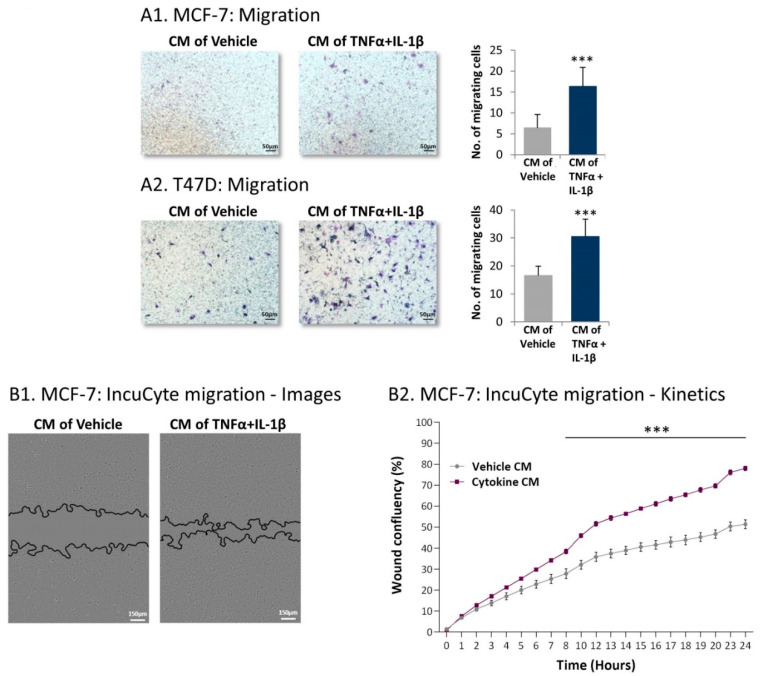
Following persistent stimulation of MSCs with TNFα + IL-1β, the resulting inflammatory CAFs release factors that promote migration of luminal-A BC cells. Human MSCs were exposed to persistent TNFα + IL-1β stimulation (concentrations as in Figure 1) or to vehicles, generally for 14–18 days. Cytokine-devoid CM were collected as described in Figure 7 and were administered to mCherry-expressing MCF-7 and T47D human luminal-A BC cells. (**A**) Tumor cell migration was determined in fibronectin-coated transwells in response to serum-containing medium. (**A1**) MCF-7 cells. (**A2**) T47D cells. Images and quantitative analyses of a representative experiment out of *n* = 3, for each cell type, are presented. *** *p* < 0.001. (**B**) MCF-7 wound closure assay, performed in IncuCyte^®^. (**B1**) Representative images taken at 24 h. Bar, 150 µm. (**B2**) The kinetics graphs of tumor cell migration demonstrate the proportion (%) of the original wound area that became covered by migrating cells at each time point. The results presented are mean ± SEM of 5 replicates for each treatment and are of a representative experiment out of *n* > 3. *** *p* < 0.001.

Further supporting the metastasis-stimulating activity of factors released by the inflammation-driven CAFs was the fact that, following treatment using cytokine-devoid CM derived from the inflammatory CAFs, both MCF-7 and T47D cells demonstrated increased migration towards serum-containing medium (Figure 9A). Moreover, kinetics analyses of wound closure assays revealed that powerful migratory activities were gained by MCF-7 cells that were exposed to cytokine-devoid CM of the inflammatory CAFs (Figure 9B) (similar analyses were not performed with T47D cells because they detached from the IncuCyte^®^ plates during scratch formation). The elevated migratory potential of the tumor cells following growth with cytokine-devoid CM of inflammation-driven CAFs was not caused by increased proliferation; rather, cancer cells treated by CM of inflammatory CAFs demonstrated reduced growth ability compared to tumor cells treated by CM of control cells (Figure S3). 

Here, it is interesting to note that, following their growth with cytokine-devoid CM from inflammatory CAFs, MCF-7 cells also demonstrated characteristics that are related to epithelial-to-mesenchymal transition (EMT) (Figure S4), a phenotype connected to increased migration [53,54]. In this case, we noted that the cytokine-devoid CM of inflammation-driven CAFs induced reduction in E-cadherin and elevated the levels of vimentin in the cancer cells; the alterations were not pronounced (Figure S4), suggesting that the tumor cells underwent a partial EMT process that, based on published articles, can support cancer progression [53].

### 2.5. Inflammation-Driven CAFs Promote Tumor Cell Migration via Ligands of Ras-Activating Receptors and via Inflammatory Chemokines 

Next, we explored the identity of the factors released by inflammation-driven CAFs, which promoted tumor cell migration. We addressed this question by blocking in the tumor cells two major signaling components that are of high importance to cancer: (1) the Ras protein, which is stimulated typically by receptor tyrosine kinases (RTKs) [55,56,57], and (2) the heterotrimeric G protein Gαi that mediates signaling using many G protein-coupled receptors (GPCRs) [58,59,60]. The signaling by Ras and by Gαi was inhibited by farnesylthiosalicyclic acid (FTS) (Salirasib) or by pertussis toxin (PTx), respectively. In these experiments, the focus was on identification of inflammatory CAF-driven factors that could affect migration of the cancer cells; thus, different inhibitors were added to the tumor cells treated by inflammatory CAF-driven CM during the time of the migration assay; the group of cancer cells treated by CM of vehicle-treated MSCs served as a baseline to determine the extent of reduction in migration caused by the inhibitors.

**Figure 10:**
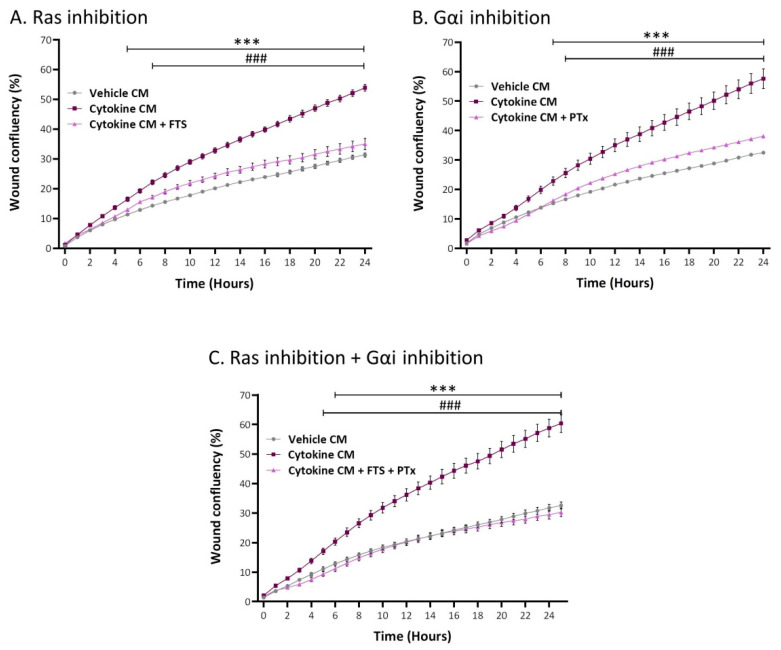
Tumor cell migration, elevated by inflammatory CAF-derived factors, is mediated through cooperativity between Ras-activating receptors and G protein-coupled receptors (GPCR) that signal via Gαi. Human MSCs were exposed to persistent TNFα + IL-1β stimulation (concentrations as in Figure 1) or to vehicles, generally for 14–18 days. Cytokine-devoid CM were collected as described in Figure 7 and were administered to MCF-7 cells in the presence of (**A**) FTS, an inhibitor of Ras; (**B**) PTx, an inhibitor of Gαi; or (**C**) both inhibitors together. Control cells were treated by the vehicles of inhibitors. Inhibitor concentrations were selected based on the considerations described in the Materials and Methods section. Cell numbers and viability were not affected by the inhibitors. Then, kinetics analyses of wound closure assays were performed in IncuCyte^®^, as described in Figure 9B. The results are presented as mean ± SEM of 8–9 replicates for each treatment. *** *p* < 0.001, for differences between tumor cells treated by CM of inflammation-derived CAFs and control CAFs. ### *p* < 0.001, for differences between tumor cells treated by the inhibitors and tumor cells that were not treated by the inhibitors. The results of a representative experiment out of *n* = 3 are presented.

The data presented in Figure 10 indicate that each of the two key signaling pathways partly mediated the migration-inducing activities of inflammatory CAF-derived factors, as indicated by significant inhibition obtained by FTS (Figure 10A) and PTx (Figure 10B) (reduction levels were in the ranges 35–84% and 39–78% in different experiments, for FTS and PTx, respectively). Moreover, we also determined that, when the two inhibitors were together in the same experiment, migration was more potently inhibited compared to each inhibitor alone and was reduced to the basal migration level demonstrated by tumor cells grown with CM of vehicle-treated cells (Figure 10C). Of note, none of the inhibitory modalities affected cell numbers or viability. These findings indicate that the factors released by the inflammation-driven CAFs induced tumor cell migration by activating receptors that signal through Ras and through GPCRs that activate cells via Gαi, acting in a cooperative manner. 

**Figure 11:**
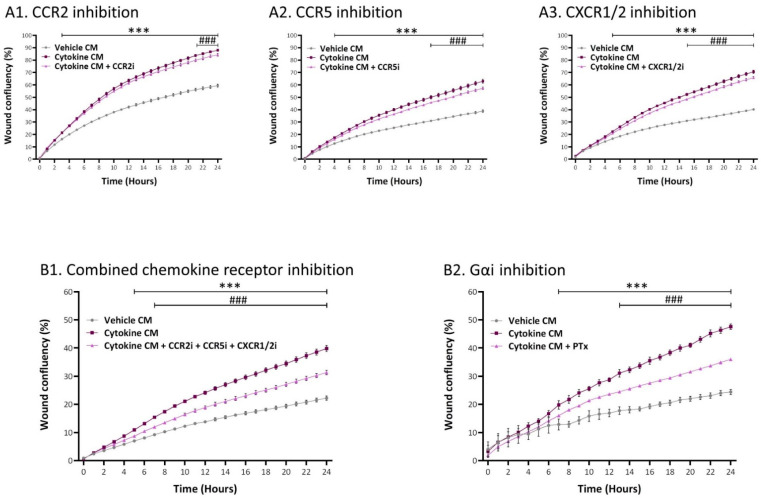
Tumor cell migration, elevated by inflammatory CAF-derived factors, is mediated through cooperativity between the chemokine receptors CCR2, CCR5, and CXCR1/2. Human MSCs were exposed to persistent TNFα + IL-1β stimulation (concentrations as in Figure 1) or to vehicles, generally for 14–18 days. Cytokine-devoid CM were collected as described in Figure 7 and were administered to MCF-7 cells in the presence of inhibitors. (**A**) Chemokine receptor inhibitors. (**A1**) CCR2i = the CCR2 inhibitor CAS 445479–97-0; (**A2**) CCR5i = the CCR5 inhibitor Maraviroc; and (**A3**) CXCR1/2i = the CXCR1/2 inhibitor Reparixin. (**B**) Combined inhibitory measures directed to chemokine receptors compared to inhibition of Gαi by PTx performed in the same experiment. (**B1**) All three chemokine receptor inhibitors together. (**B2**) Inhibition by PTx. In all treatments, cell numbers and viability were not affected by the inhibitors. Kinetics analyses of wound closure assays were performed in IncuCyte^®^, as described in Figure 9. The results are presented as mean ± SEM of 6–9 replicates for each treatment. *** *p* < 0.001, for differences between tumor cells treated by the CM of inflammation-derived CAFs and control CAFs. ### *p* < 0.001, for differences between tumor cells treated by the inhibitors and tumor cells that were not treated by the inhibitors. The results of a representative experiment out of *n* = 3 are presented (with the exception of *n* = 2/3 in Figure 9(A1)).

In previous parts of the study, we demonstrated that specific chemokines—CCL2, CCL5, and members of the ELR+ CXC chemokine family (CXCL1, CXCL2, CXCL6, and CXCL8)—were prominently upregulated at the gene and protein levels in the inflammatory CAFs (Figure 5C). Because the receptors of these chemokines—CCR2, CCR5, and CXCR1/2, respectively—signal via Gαi [61,62,63], in a new set of experiments, we targeted these receptors using specific inhibitors. Here, we used CAS 445479-97-0 to block CCR2; Maraviroc was employed to block CCR5; and Reparixin was used to inhibit CXCR1/CXCR2. The findings of Figure 11A indicate that each of these three inhibitors had a significant but minimal ability to decrease the migration rate of tumor cells that were exposed to CM of the inflammatory CAFs. However, when all three inhibitors were combined in the same experiment (without affecting cell numbers or viability; additive effects were revealed and a pronounced inhibition of tumor cell migration was noted (Figure 11(B1)). In parallel, because CCR2, CCR5, and CXCR1/2 signal by activating Gαi, in this set of experiments, we included PTx again; this step was taken in order to enable proper comparison in the same experiment of the reduction level obtained by inhibitors of all three chemokine receptors together to the levels obtained by PTX, which is a general inhibitor of all Gαi-mediated responses. The findings of Figure 11B demonstrate similar inhibitory levels for PTx and for inhibitors directed to CCR2 + CCR5 + CXCR1/CXCR2 together.

**Figure 12:**
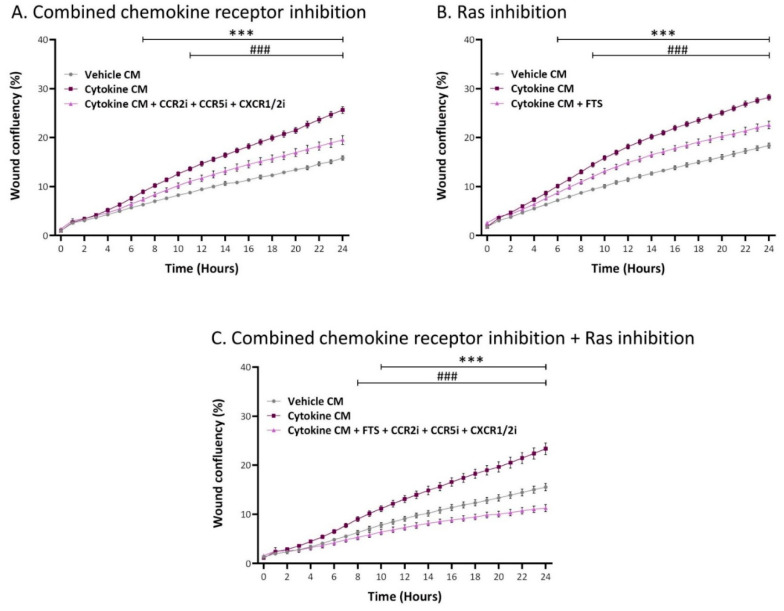
Tumor cell migration, elevated by inflammatory CAF-derived factors, is mediated through cooperativity between Ras-activating receptors and chemokine receptors. Human MSCs were exposed to persistent TNFα + IL-1β stimulation (concentrations as in Figure 1) or to vehicles, generally for 14–18 days. Cytokine-devoid CM were collected as described in Figure 7 and were administered to MCF-7 cells in the presence of inhibitors. In the same experiment, different inhibitory conditions were used: (**A**) combined inhibition of CCR2, CCR5, and CXCR1/2 together, as in Figure 11. (**B**) Inhibition of Ras-mediated signaling by FTS. (**C**) Combined inhibitory measures directed to chemokine receptors and Ras-mediated signaling. In all treatments, cell numbers and viability were not affected by the inhibitors (except for the inhibitory treatment included in part C, where 15–25% decrease in cell numbers was noted). Kinetics analyses of wound closure assays were performed in IncuCyte^®^, as described in Figure 9. The results are presented as mean ± SEM of 8–9 replicates for each treatment. *** *p* < 0.001, for differences between tumor cells treated by the CM of inflammation-derived CAFs and control CAFs. ### *p* < 0.001, for differences between tumor cells treated by the inhibitors and tumor cells that were not treated by the inhibitors. The results of a representative experiment out of *n* = 3 are presented.

Therefore, the data provided above suggest that CCL2, CCL5, and ELR + CXC chemokines—that are upregulated in the CM of the inflammation-driven CAFs (Figure 5(C2))—are the factors that were released by the inflammation-driven CAFs and promoted tumor cell migration by activating their cognate Gαi-stimulating receptors (Figure 11). To demonstrate that indeed these chemokine receptors accounted for the entire Gαi-mediated response that cooperated with Ras-mediated signaling (Figure 10), a new set of experiments was performed. In this set, the first treatment included all three inhibitors of chemokine receptors combined; the second treatment included FTS alone; and the third treatment included FTS + chemokine receptor inhibitors, together. This approach was taken in order to determine whether the Ras-activating receptors and the chemokine receptors complement each other and the joint administration of their inhibitors leads to complete inhibition of cancer cell migration. Indeed, the data of Figure 12 indicate that the Ras-activated pathways and the chemokine receptor-mediated pathways acted in cooperativity, leading to full blockade of tumor cell migration (a 15–25% reduction in tumor cell numbers was noted in the treatment of Figure 12C in different experiments). 

Taken together, the above results indicate that the inflammatory CAFs—obtained following persistent stimulation of MSCs by inflammatory cytokines—release ligands that stimulate Ras-activating receptors and also inflammatory chemokines that activate CCR2, CCR5, and CXCR1/2, which jointly act on luminal-A cells to increase their migratory potential. 

## 3. Discussion

Stromal cells constitute a major part of the TME and have cardinal roles in regulating tumor progression. Within this context, many different pro-malignancy roles were attributed to CAFs, such as induction of stemness, proliferation, chemoresistance, and invasion of tumor cells; in parallel, CAFs act on elements of the TME, providing support to the cancer cells by elevating matrix remodeling, angiogenesis, and immune suppression [11,12,14,29,30,31,32,33,34,35].

CAFs can be derived from many different sources, including resident tissue fibroblasts, adipose MSCs, and BM-MSCs [14,29,31,33,64]. The mechanisms that drive the conversion of MSCs to CAFs and shape stromal cell plasticity have been at the center of extensive research. Recent studies indicated that prolonged stimulation of MSCs by tumor-derived factors could lead to their conversion to CAFs, as did TME-derived factors such as transforming growth factor β, CXCL12, and osteopontin [29,30,65,66]. 

The findings of the current study point to the strong impact of continuous inflammatory stimulus, which is driven by TNFα and IL-1β—that mediate chronic tumor inflammation—on the process of MSC-to-CAF conversion. In our study, we provide evidence to three novel observations: (1) persistent stimulation of MSCs by inflammatory cytokines induces MSC-to-CAF conversion; (2) persistent stimulation by inflammatory cytokines has a much more robust impact on the MSCs than short-term cytokine stimulation and leads to the generation of inflammatory CAFs; and (3) factors that are released by inflammation-driven CAFs promote the migratory abilities of luminal-A breast tumor cells by inducing the activation of Ras-activating receptors, together with the chemokine receptors CCR2, CCR5, and CXCR1/2.

Thus, although interactions between tumor-related inflammation and stromal cells were reported previously by our studies [43,44,45,67] and others [40,41,42,68,69,70,71,72], our current investigation is the first to show that persistent stimulation of MSCs by the potent pro-inflammatory cytokines TNFα and IL-1β leads to their conversion to cancer-supporting inflammatory CAFs. The identity of the cells as inflammatory CAFs was based on a gradual analysis that we performed, demonstrating that, following continuous stimulation of MSCs by TNFα + IL-1β, the resulting cells expressed a CAF-like morphology, thus showing that they expressed typical CAF markers and functions, and ultimately that the resulting CAFs expressed the phenotype of inflammatory CAFs. The identification of the cells as inflammatory CAFs is of interest in view of recent reports that have provided evidence of the immense plasticity of CAFs [36,37,38,39]. For example, inflammatory CAFs have been identified in PDAC by the Tuveson group, demonstrating that these cells are located in desmoplastic areas of the tumors, are enriched with the expression of pro-inflammatory mediators such as IL-6 and LIF and are generated by IL-1 signaling [50,51].

Moreover, our findings illustrate the importance of continuous stimulation by the pro-inflammatory cytokines TNFα + IL-1β, known as metastasis-promoting factors (TNFα [16,17,18] and IL-1β [19,20,21]) over short-term stimulation. The advantages provided by the persistent cytokine stimulation of MSCs were demonstrated in our study by the extensive CAF-like morphology gained by the cells, which was not observed following short-term stimulation by the cytokines; furthermore, the RNAseq analyses that we performed clearly pointed at much more robust alterations in the transcriptome induced by continuous TNFα + IL-1β stimulation compared to short-term stimulation. Thus, our study provided evidence to new characteristics that were acquired by BM-MSCs following persistent cytokine stimulation, which were not reported previously, particularly not after short-term stimulation using TNFα and/or IL-1β. Published investigations from our lab and others demonstrated that, under brief stimulatory conditions, generally of up to 48 h, the impact of these cytokines was reflected by increased expression of pro-inflammatory mediators (such as IL-6 and PGE2) [48], chemokines and their receptors (e.g., CCL2, CXCL8, CXCL10, CXCR4, and more) [40,43,45,69,73], adhesion molecules [74,75], angiogenic factors, and growth factors (VEGF, FGF2, IGF-1, and HGF) [40,76] and by affecting the immune system [77,78]. However, these studies did not demonstrate that the MSCs underwent extensive morphological changes and gained the phenotype and functional properties of inflammatory CAFs upon short-term TNFα and/or IL-1β stimulation. 

Therefore, our study extends much of the current knowledge on the impact of TNFα + IL-1β on MSCs and reveals a new phenotype of inflammatory CAF that is gained by MSCs following continuous stimulation by pro-inflammatory mediators. Furthermore, this phenotype is accompanied by the ability of the inflammatory CAFs to release factors promoting the migration of luminal-A BC cells. These observations agree well with emerging reports on the impact of long-term stimulations in elevating tumor-supporting events in other cancer systems [79,80,81]. This approach is particularly relevant in our study because TNFα and IL-1β were found to be expressed persistently in breast tumors, to exert powerful and causative tumor-promoting roles, and to be significantly associated with poor prognosis in patients [16,17,18,19,20,21,22,23,24,25,26,27]. Here, it is interesting to consider the actual concentrations of the two cytokines in the tumor and the way they exert their impacts in a dose-dependent manner; it is possible, for example, that the relatively high dose of TNFα (50 ng/mL) that was required in order to obtain MSC-to-CAF conversion in our study represents non-physiological activities of the cytokine, which are beyond its typical roles in pathogen-related inflammation.

In the current study, we actually demonstrate that the inflammatory cytokines could induce the conversion of MSCs to inflammatory CAFs independently of any of the other elements that were previously reported as regulators of this process. The inflammatory CAFs derived from TNFα + IL-1β persistently stimulated MSCs, released high levels not only of IL-6 and LIF but also of many other pro-inflammatory mediators, with a definite signature of inflammatory chemokines that were secreted by the cells. This chemokine signature is of great importance because the chemokines involved were previously characterized as potent metastasis-supporting factors and were found to affect MSCs in BC and in other malignant diseases as well. However, in the current study, we exemplified a new trait of these chemokines as they were secreted by inflammation-driven CAFs and mediated their ability to promote the migratory functions of luminal-A BC cells. 

Of note, the inflammatory chemokines that were highly secreted by the inflammation-driven CAFs obtained following consistent MSC stimulation using TNFα + IL-1β—CCL2, CCL5, and ELR+ CXC chemokines such as CXCL1 and CXCL8—were found to promote the metastatic potential of cancer cells, to elevate angiogenesis, and to recruit to the tumor site leukocytes expressing tumor-promoting activities; these leukocytes include mainly myeloid cells that contribute to cancer-associated inflammation, such as monocytes that are recruited by CCL2 and CCL5 and neutrophils that are attracted to the tumor site by CXCL8 and other members of the ELR+ CXC subgroup [82,83,84,85,86,87,88,89]. Then, the leukocytes, in turn, can further contribute to disease progression by releasing growth factors, angiogenic factors, and pro-inflammatory cytokines, which can act further to promote metastatic cascades.

Thus, our findings propose the existence of a previously unreported vicious cycle in which chronic inflammation leads to MSC-to-CAF conversion and the resulting inflammatory CAFs promote the migratory potential of the cancer cells. Additionally, it is possible that the factors released by the inflammatory CAFs, primarily the chemokines, may potentiate the recruitment of myeloid cells to the tumor and thus may promote the inflammatory nature of the TME. Furthermore, these deleterious interactions between the inflammatory CAFs and the cancer cells may be further potentiated if the two cell types interact by physical contacts; this possibility is supported by our recent study of triple-negative BC cells in which we found that Notch-mediated signaling increased the pro-malignancy effects of tumor-stroma interactions [45,90]. 

The vicious cycle that takes place when luminal-A BC cells communicate with stromal cells under inflammatory conditions points at the need to halt inflammation–stroma interactions in order to prevent cancer progression, setting inflammatory mediators as potential targets for treatment. As such, one should consider using anti-inflammatory drugs as new therapeutic modalities that can be introduced to the treatment of luminal-A BC patients. Such drugs may break the vicious cycle driven by inflammation–stromal interactions and may reduce tumor progression. Targeting chronic inflammation has been recently shown to prevent cancer development, as demonstrated by the use of non-steroidal anti-inflammatory drugs (NSAIDs). Prolonged use of aspirin, an NSAID that inhibits cyclooxygenase 1 (COX1) and COX2 and thus decreases inflammation, was recently reported to significantly reduce the incidence of cancer mainly in colorectal cancer and in other malignancies, including BC [91,92]. However, it is currently not known whether aspirin or other NSAIDs can prevent tumor progression once the process has begun.

Our data suggest that inflammatory processes in luminal-A tumors need to be targeted from the very initial stages of tumor growth, once the primary tumor is enriched by inflammatory factors and is populated by BM-derived MSCs. We propose that general inhibitors of inflammation such as NSAIDs or inhibitors of specific pro-inflammatory cytokines such as TNFα and IL-1β—that are constantly present at the tumor site from the time of malignant transformation and onwards—should be considered for the treatment of BC patients. Inhibitors of TNFα and IL-1β are currently used in the clinic in the treatment of inflammatory diseases [93,94,95] and may have many advantages when introduced to cancer therapy at the stage of tumor diagnosis. In this case, MSCs that were recruited to the tumor site from the BM will remain as is and will not convert into inflammatory CAFs that release a large variety of metastasis-promoting factors, which act on the cancer cells themselves and on the TME. 

## 4. Materials and Methods

### 4.1. Cell Growth and Stimulation by Cytokines

Human luminal-A MCF-7 cells (from ATCC) and T47D cells (provided by Dr. Keydar, who generated the cell line [96]) were grown in Dulbecco’s Modified Eagle’s Medium (DMEM) medium, supplemented with 10% fetal bovine serum (FBS), 2% L-glutamine, and 1% penicillin-streptomycin-amphotericin solution (all from Biological Industries, Beit Ha’emek, Israel). Validated human primary BM-derived MSCs were obtained from Lonza (#PT-2501; Basel, Switzerland), were kept in culture for up to nine passages, and were grown as previously described [45]. All findings were validated with MSCs of two healthy female donors or more (except for a very small number of cases, where indicated). 

Based on preliminary titration studies performed in our lab and on other in vitro reports using TNFα [97,98,99] and IL-1β [100,101], MSCs were stimulated by recombinant human (rh) TNFα (50 ng/mL; #300-01A, PeproTech, NJ, USA) and/or rhIL-1β (0.5 ng/mL; #200-01B, PeproTech). Control cells were treated by the vehicles of the cytokines. Based on kinetics analyses (Figure 1A), MSCs were generally exposed to persistent exposure to the cytokines/vehicles for 14–18 days, with similar results obtained for studies within this time range. In other studies, the MSCs underwent short-term stimulation of 48 h with the cytokines. Of note, persistent stimulation for 18 days by TNFα + IL-1β, in which the TNFα dose was reduced to 10 ng/mL, showed only a partial ability to modify the morphology of MSCs (compared to 50 ng/mL TNFα; Figure S5). 

### 4.2. Morphological Assessment and IN Cell Analyses

In morphological studies, MSCs were treated by TNFα + IL-1β/vehicles for up to 19 days. At different time points, the cells were photographed using light microscopy. In parallel, in IN Cell studies, MSCs were cultured for 14–18 days in 384-well microplates (#781091, Greiner Bio-One, Kremsmunster, Austria) under automated High-Throughput Screening conditions (Freedom EVO 200 robot, Tecan Group Ltd., Männedorf, Switzerland). The cells were stained and imaged by the IN Cell Analyzer 2200 automated microscope (GE Healthcare, Arlington Heights, IL, USA) at X20 magnification (40 replicates/treatment). Cell and nucleus sizes were determined by cytoplasmic and nuclear staining using calcein (#C3100MP, Thermo Fisher Scientific, Waltham, MA, USA) and Hoechst (#H1399, Thermo Fisher Scientific), respectively. Following image acquisition, high-content image stacks were analyzed using the IN Cell developer Toolbox 1.9.3 (GE Healthcare) producing an output based on comparative fluorescence intensity.

In other studies, luminal-A BC cells were grown with CM collected from MSCs that were persistently treated with TNFα + IL-1β/vehicles. In this case, the MSCs were stimulated by TNFα + IL-1β, generally for 14–18 days; then, they were replenished for 48 h with fresh medium (containing 0.5% serum) that did not contain exogenously added cytokines. These cytokine-devoid CM were collected, filtered (0.45 μm), X2 concentrated (Amicon Ultra-15 Centrifugal Filter Unit, #UFC900324, Merck, Darmstadt, Germany), and administered to BC cell cultures. Tumor cell characteristics were imaged by confocal analyses (see below) and by the IN Cell Analyzer 2200 (GE Healthcare), as described above.

### 4.3. Transcriptome Analyses 

To determine transcriptome alterations upon short-term (48 h) and persistent cytokine stimulation (14 days), with each cytokine alone or together, genome-wide expression analysis was performed on MSCs in two sets of experiments (with cells of the same donor): Set 1: MSCs were stimulated by TNFα and IL-1β or by vehicles for 14 days (persistent stimulation). Set 2: MSCs were stimulated by TNFα and/or IL-1β or by vehicle controls for 48 h (short-term stimulation) or for 14 days (persistent stimulation). Each set of experiments included 3 biological repeats of stimulated cells and of vehicle-treated cells.

Total RNA was isolated using miRNeasy Mini Kit (#217004, Qiagen, Hilden, Germany) and then submitted for RNAseq at the Israel National Center for Personalized Medicine (INCPM). The libraries were prepared using in-house protocol, and the samples were sequenced on 8 lanes of the Illumina HiSeq-2500 machine, using the Single-Read 60 protocol. The output was ≈18 million reads per sample. Raw reads were preprocessed by trimming Illumina adapters (AGATCGGAAGAGCACACGTCTGAACTCCAGTCAC), and poly A and poly T tails, and by trimming edges of reads with quality below 10 using Cutadapt v1.8.3. Reads with lengths below 30 were discarded. The processed reads were mapped to human genome, GRCh38, using STAR v2.4 [102] and then the number of reads that were mapped to each gene was calculated using HTSeq-count, v0.6.1p1 [103] with intersection-strict mode. The gene annotation used for the counting was Ensemble annotation for GRCh38, release 83. Differential expression was carried out using Deseq2 [104]. Adjusted *p*-values (padj) were taken from Deseq correction for multiple testing, which is based on Benjamin–Hochberg false discovery rate (FDR). Bioinforamtics analyses were performed at the INCPM: genes that were differentially expressed in the cytokine-stimulated cells compared to vehicle-treated cells were filtered using a fold change (FC) cutoff of FC > 2/FC < 0.5, padj < 5 × 10^−10^. Then, these genes were analyzed by the INGENUITY program in order to identify affected disorders. The transcriptome data discussed in this publication were deposited in NCBI’s Gene Expression Omnibus [105] and are accessible through GEO Series accession number GSE161762.

### 4.4. Confocal Analyses

MSCs that were persistently treated by TNFα + IL-1β/vehicles, generally for 14–18 days, were subjected to confocal analyses after adhering to glass for additional 2 days in the presence of the cytokines/vehicles. The study was performed with primary antibodies directed to vimentin (#sc-6260, Santa Cruz Biotechnology, Santa Cruz, CA, USA), FAP (#sc-135069, Santa Cruz Biotechnology), or αSMA (#A2547, Sigma-Aldrich, Saint Louis, MO, USA). Secondary antibodies included Alexa 647-conjugated goat anti mouse antibody (#115-606-146, Jackson ImmunoResearch Laboratories, West Grove, PA, USA), DyLight 550-conjugated donkey anti rabbit antibody (#ab96920, Abcam, Cambridge, UK), or Fluorescein isothiocyanate (FITC)-conjugated goat anti mouse antibody (#115-095-003, Jackson ImmunoResearch Laboratories), as required. Fluorescent signals were quantified by the ImageJ program (National Institutes of Health, Bethesda, MA, USA) in ≥5 fields for each treatment in all experiments, and the findings of the representative experiment are presented.

In titration analysis of TNFα doses, MSCs were stimulated by 10 ng/mL TNFα vs. 50 ng/mL TNFα in the presence of IL-1β (0.5 ng/mL) for 18 days. After culturing the cells on glass for an additional 2 days in the presence of the cytokines/vehicles, their morphology was determined by FITC-conjugated phalloidin staining (#P-5282, Sigma-Aldrich) that stains actin filaments. 

In other studies, cytokine-devoid CM were collected from MSCs that were persistently treated with TNFα + IL-1β/vehicles (prepared as described above) and administered to BC cultures. After 2–3 days, actin filaments were detected in the tumor cells by FITC-conjugated phalloidin staining (as above) and cell nuclei were detected by Hoechst staining (#B2261, Sigma-Aldrich). 

In all cases, the cells were analyzed by confocal microscopy (Leica SP8) at X40 magnification. 

### 4.5. Secretome Analyses

To perform secretome analyses, MSCs were persistently treated by TNFα + IL-1β or by vehicles for 18–19 days in three biological repeats. The cells were washed and grown for additional 48 h in cytokine, serum, and phenol red-devoid media. CM were filtered (0.45 μm) and then submitted for mass spectrometry and bioinformatics analysis at the INCPM. The samples were concentrated, and the proteins were extracted, reduced, and alkylated and then were subjected to trypsin digestion. Following a desalting step, samples were loaded using split-less nano-Ultra Performance Liquid Chromatography (10 kpsi nanoAcquity; Waters, Milford, MA, USA). The peptides were then separated using a T3 HSS nano-column and eluted into tribrid Orbitrap Fusion Lumos mass spectrometer (Thermo Fisher Scientific). Data were acquired in data-dependent acquisition (DDA) mode. Raw data were processed with MaxQuant v1.6.0.16 using the default parameters (including FDR correction) except the following: the enzyme was set to trypsin, LFQ (Label-Free Quantification) min. ratio count = 1, peptides for quantification = unique and match between runs was enabled. The data were searched with the Andromeda search engine against the Uniprot human proteome database (March 2018 version) appended with common lab protein contaminants. The following modifications were defined for the search: carbamidomethylation of C as a fixed modification and oxidation of M; N-term acetylation and carbamylation, and hydroxy-P as variable ones. The LFQ intensities were extracted and used for further calculations in Perseus v1.6.0.7. Decoy hits were filtered out, as well as proteins that were identified on the basis of a modified peptide only. The LFQ intensities were log transformed, and only proteins that had at least 2 valid values in at least one experimental group were kept. Missing values were imputed, and the data underwent further statistical analysis using a Student’s t-test. The FC for each comparison was calculated based on the geometric mean of the calculated LFQ intensities for each experiment. Bioinformatics analyses were performed at the INCPM: proteins that were differentially expressed in the cytokine-stimulated cells compared to vehicle-treated cells were filtered using a cutoff FC ≥ 2/FC ≤ 0.5, *p* < 0.05. Then, the INGENUITY program was used to identify affected disorders. The mass spectrometry proteomics data were deposited to the ProteomeXchange Consortium [106] via the partner repository with the dataset identifier PXD022635.

### 4.6. Collagen Contraction Assays 

MSCs were persistently treated with TNFα + IL-1β/vehicles, generally for up to 14–18 days. Afterwards, the cells were cultured in 24-well plates in collagen-containing medium (0.5 mg/mL; #345236, Corning, Corning, NY, USA) with or without TNFα + IL-1β and were incubated at 37 °C for 1 h for collagen gel polymerization. Then, the collagen gels were gently released from the edges of the wells using a sterile tip and the plates were returned to incubation for up to 13 day; exogenous cytokines were not replenished during this incubation period. Images of the gels were taken from the top of the plates at different time points. Contraction quantification was performed using ImageJ program. 

### 4.7. Spheroid Formation Assays

Spheroid formation assays were performed in conditions that prevent the cells from adhering to substrate, thus forming distinct spheroids. In the case of mCherry-expressing MCF-7, the cells were incubated for 48 h with cytokine-devoid CM of MSCs that were persistently treated with TNFα + IL-1β or with CM of vehicle-treated MSCs (prepared as described above). Then, the tumor cells were plated in DMEM/F12 medium containing B-27 supplement, basic fibroblast growth factor, epidermal growth factor, and insulin in the absence of CM. After 2 days, tumor-spheroids were formed and were transferred for 8 days to fresh cytokine-devoid CM of MSCs (that were persistently treated beforehand with TNFα + IL-1β or with vehicles). 

In the case of mCherry-expressing T47D cells, cytokine-devoid CM of MSCs (that were persistently treated beforehand with TNFα + IL-1β/vehicles) were added to the tumor cells for 72 h. Then, the cells were plated in fresh cytokine-devoid CM, which included the abovementioned supplements to enable spheroid formation, for 4 days. 

In both cell types, the cells were photographed using a fluorescence microscope at X4 magnification. Based on fluorescent signals, the size of spheroids was quantified by the ImageJ program in ≥7 fields for each treatment. 

### 4.8. Flow Cytometry Analyses

MSCs were persistently treated by TNFα + IL-1β/vehicles, generally for 14–18 days. Cytokine-devoid CM were collected (prepared as described above) and were added to MCF-7 cells for 72 h. 

The extracellular expression of E-cadherin was determined by flow cytometry using Allophycocyanin (APC)-conjugated anti human E-cadherin mouse IgG1 antibody (#324107, Biolegend, San Diego, CA, USA). To determine the intracellular expression of vimentin by flow cytometry, the cells were fixed and permeabilized using 100% methanol for 10 min at −20 °C. Then, the expression of vimentin was detected by anti human vimentin mouse IgG1 antibody (#sc-6260, Santa Cruz Biotechnology) followed by Alexa 647-conjugated goat anti-mouse antibody (#115-606-146, Jackson ImmunoResearch Laboratories). Baseline staining was determined by relevant isotype-control antibodies. The fluorescence level was determined using Flow Cytometer S100EXi (Stratedigm, San Jose, CA, USA) with CELLCAPTURE software (Stratedigm) and analyzed by FLOWJO V10 (BD biosciences, OR, USA).

### 4.9. Transwell and IncuCyte Migration Assays

MSCs were persistently treated by TNFα + IL-1β/vehicles, generally for 14–18 days. Serum- and cytokine-devoid CM were collected (prepared as described above) and were added to mCherry-expressing MCF-7 and T47D cultures for 48 or 72 h, respectively. Then, tumor cells were plated in transwells with 8 μm pore membranes (#3422, Corning) for 21.5–24 h in fresh cytokine-devoid CM. The bottom wells contained fresh cytokine-devoid CM supplemented with 10% FBS. The cells that migrated to the lower part of the transwells were fixed and stained with Hemacolor (#111661, Merck). The cells were photographed using light microscopy and counted at multiple fields. 

In parallel, migration studies were performed using an IncuCyte^®^ Live-Cell Imaging System. Here, cytokine-devoid CM obtained from MSCs (that were persistently stimulated beforehand with TNFα + IL-1β/vehicles; prepared as described above) were added to MCF-7 cells that were plated in IncuCyte^®^ ImageLock 96-well plates (#4379, Essen BioSciense, Ann Arbor, MI, USA). After scratching the cells in the middle of the wells, kinetics analyses of tumor cell migration were performed for 24 h. When appropriate, the following inhibitors were added during the IncuCyte^®^ migration assay: for Ras, farnesylthiosalicyclic acid (FTS, Salirasib; 7 µM; #SML1166, Sigma-Aldrich); for Gαi, Pertussis Toxin (PTx; 0.1–0.2 µg/mL; #516560, Calbiochem, Merck); for CCR2, CAS 445479-97-0 (0.02–8 µM; #227016, Calbiochem, Merck); for CCR5, Maraviroc (30 µM; #14641, Cayman Chemical, Ann Arbor, MI, USA); and for CXCR1/2, Reparixin (30–40 µM; #A12383, ADOOQ Bioscience, Irvine, CA, USA). Inhibitor concentrations were determined in preliminary analyses that guaranteed that they did not affect tumor cell proliferation or viability (except for combined treatment with FTS and the three chemokine receptor inhibitors (15–25% reduction, in different experiments)). The final concentrations of the inhibitors that were selected for use were within the range used in other studies and within the range recommended by the manufacturers. 

### 4.10. Determination of Cell Number and Viability

To determine the effect of persistent stimulation with TNFα + IL-1β on MSC proliferation, the cells were plated at equal numbers as of day 7 of cytokine/vehicle treatment. Following stimulation for a total of 14–18 days, cell numbers were determined. Alternatively, kinetics analysis of MSC proliferation was performed. To this end, MSCs were stimulated by the cytokines or exposed to vehicles for 7 days. Then, the cells were plated in equal numbers and their proliferation at days 10, 14, and 18 after initiation of stimuli was determined. In all these assays, cell numbers were determined by trypan blue exclusion assay in ≥2 replicates/treatment.

To determine the effect of cytokine-devoid CM collected from MSCs (that were persistently treated beforehand with TNFα + IL-1β/vehicles; prepared as described above) on the proliferation of luminal-A BC tumor cells, the cancer cells were plated in equal numbers and then were grown with CM of each of the two MSC types for 40 h. Then, tumor cell proliferation was determined by counting the cells by trypan blue exclusion assay in ≥6 replicates/treatment. 

To determine the effects of FTS, PTx, CAS 445479-97-0, Maraviroc, Reparixin, and/or their combinations on tumor cell numbers and viability, three independent experiments were performed. Here, the inhibitors were added to MCF-7 cells (in media containing 0.5% serum) for 24 h, adhering to the time period that the cells were exposed to the inhibitors in the IncuCyte experiments. Then, the cells were counted by trypan blue exclusion assay in ≥2 replicates/treatment. 

### 4.11. Statistical Analyses 

Statistical analyses were performed by two-tailed unpaired Student’s *t*-tests, or two-way ANOVA in collagen contraction assays and IncuCyte studies. Statistical analyses of the transcriptome and secretome analyses are described in their relevant sections. 

## 5. Conclusions

In this study, we addressed the process of MSC-to-CAF conversion, which is a cardinal aspect of tumor cell biology. We provided novel evidence on the deleterious impact of tumor-related chronic inflammation, manifested by its ability to induce the gradual changeover of mesenchymal stem cells—that reach the tumor site from the bone marrow—to deleterious CAFs that carry an inflammatory phenotype. As such, these inflammatory CAFs promoted pro-metastatic activities in the cancer cells, enabling them to disconnect from each other and to migrate in a highly efficient manner.

These findings contribute to the global efforts aimed at discovering the regulatory events leading to high CAF presence in tumors and are instrumental in terms of future drug design. These observations can set the ground for the use of inflammation-inhibiting modalities in cancer, reducing the levels of metastasis-supporting CAFs at the TME and thus providing improved therapeutic modalities in BC.

## Data Availability

Please see in Materials and Methods section.

## Supplementary Materials

The following are available online at https://www.mdpi.com/2072-6694/13/6/1472/s1: Figure S1: Genes that are expressed in low levels in BM-MSC-derived CAFs, are down-regulated upon the conversion of BM-MSCs to inflammatory CAFs, Figure S2: In time-dependent analysis of MSC stimulation by TNFα+IL-1β, the stimulated cells demonstrate increased proliferation rate at all time points, Figure S3: Following persistent stimulation of MSCs with TNFα+IL-1β, the resulting inflammatory CAFs release factors that decrease the proliferation rate of luminal-A BC cells, Figure S4: Following persistent stimulation of MSCs with TNFα+IL-1β, the resulting inflammatory CAFs release factors that induce a partial EMT phenotype in luminal-A BC cells, Figure S5: Titration of TNFα concentration required for induction of CAF-like morphology in persistently-stimulated MSCs, Table S1: Persistent stimulation of MSCs with TNFα+IL-1β: 60 top up-regulated genes, Table S2: Persistent stimulation of MSCs with TNFα+IL-1β: 60 top down-regulated genes, Table S3: Persistent stimulation of MSCs with TNFα+IL-1β: 60 top up-regulated secreted proteins, Table S4: Persistent stimulation of MSCs with TNFα+IL-1β: 60 top down-regulated secreted proteins.
