# Supplementary material for: Persistent Inflammatory Stimulation Drives the Conversion of MSCs to Inflammatory CAFs That Promote Pro-Metastatic Characteristics in Breast Cancer Cells
Source: Cancers (Basel). 2021 Mar 23;13(6):1472. doi: 10.3390/cancers13061472 (PMC8004890; doi:10.3390/cancers13061472)
Supplement: Supplementary file 1 [file cancers-13-01472-s001.zip › Supplementary Tables 1-4-28.2.21.pdf]

Supplementary Table S1

	Gene	Fold change	padj
1	MIR3142HG	1.18E+04	2.1E-06
2	GPR4	8.61E+03	4.81E-06
3	CXCL5	6.90E+03	5.3E-271
4	BCL2A1	6.10E+03	1.59E-05
5	ADAMTS18	5.70E+03	2.69E-05
6	CXCL6	4.28E+03	1.4E-213
7	MMP3	3.52E+03	8.8E-107
8	IL1B	2.76E+03	4.1E-113
9	CSF3	2.32E+03	7.74E-26
10	MMP8	2.25E+03	0.000142
11	CXCL3	2.06E+03	8.9E-104
12	CXCL1	1.86E+03	7.11E-68
13	CXCL8	1.83E+03	3.9E-55
14	PPBP	1.71E+03	0.000231
15	CCL20	1.60E+03	4.24E-12
16	MMP9	1.54E+03	0.000738
17	HEPHL1	1.32E+03	4.63E-30
18	PF4	1.25E+03	0.000458
19	SOX17	1.14E+03	0.004705
20	IL1A	1.03E+03	7.05E-57
21	CSF2	1.03E+03	1.26E-37
22	LCP1	9.13E+02	5.22E-10
23	MMP13	8.99E+02	0.00096
24	SPRR2A	8.90E+02	0.001049
25	EHF	8.14E+02	4.12E-35
26	MMP1	8.08E+02	6.01E-37
27	SLC7A2	7.95E+02	3.26E-24
28	SERPINA9	7.79E+02	6.17E-90
29	CCL7	7.22E+02	0.001911
30	SNCB	6.56E+02	0.002442

	Gene	Fold change	padj
31	MMP10	5.68E+02	0.002205
32	GPR84	5.33E+02	0.002733
33	SERPINB4	5.29E+02	1.23E-08
34	CD93	4.97E+02	0.003351
35	DNER	4.92E+02	4.23E-48
36	C15orf48	4.51E+02	5.29E-29
37	CAMK1G	4.51E+02	0.005553
38	NKX2-6	4.43E+02	0.003989
39	LINC00996	4.41E+02	0.003518
40	LINC01050	4.36E+02	0.003797
41	CTD-3128G10.7	4.23E+02	0.003642
42	SLAMF7	4.08E+02	0.00353
43	CITF22-49D8.1	3.93E+02	0.004517
44	CCL3	3.90E+02	3.17E-08
45	LINC00519	3.89E+02	0.004586
46	RP11-13A1.1	3.87E+02	0.004932
47	MMP12	3.86E+02	8.43E-17
48	RP11-753N8.1	3.84E+02	0.005071
49	CTD-2369P2.8	3.80E+02	0.004825
50	SLC26A9	3.64E+02	0.005356
51	PI3	3.61E+02	0.006541
52	SPSB4	3.49E+02	0.005392
53	DSCAM	3.36E+02	0.006141
54	AZGP1	3.35E+02	0.005737
55	CXCL2	3.30E+02	4.14E-100
56	SERPINA12	3.29E+02	0.006155
57	MUC13	3.25E+02	0.018663
58	CDCP1	3.20E+02	2.87E-152
59	GNA15	3.07E+02	0.007575
60	KDR	3.03E+02	0.006941

Persistent stimulation of MSCs with TNFα+IL-1β: 60 top up-regulated genes

Human MSCs were exposed to persistent TNFα+IL-1β stimulation (concentrations as in Fig. 1) or to vehicles for 14-18 days. mRNA expression was determined by transcriptome analyses performed with 3 independent biological repeats. Differentially expressed genes were determined as those having padj<0.05 values. The Table presents the 60 top up-regulated genes following TNFα+IL-1β stimulation, compared to treatment by vehicle.

Supplementary Table S2

	Gene	Fold change	padj
1	COL14A1	1.12E-02	9.61E-14
2	HR	1.12E-02	1.91E-11
3	TLX2	1.11E-02	0.038303508
4	CTC-537E7.1	1.10E-02	0.045325616
5	LINC01013	1.10E-02	3.73E-08
6	CRLF1	1.02E-02	3.67E-13
7	FEM1AP2	1.01E-02	0.038141208
8	LAMP5	1.01E-02	2.11E-08
9	LYVE1	1.01E-02	0.03803195
10	RP3-495K2.2	9.94E-03	0.038268869
11	INPP5D	9.09E-03	0.030315785
12	B3GALT2	9.05E-03	9.81E-35
13	GJA5	9.01E-03	0.030138004
14	FLG	8.80E-03	2.99E-34
15	SLC1A7	8.50E-03	2.46E-05
16	PLCE1-AS1	8.03E-03	1.74E-56
17	CPZ	7.76E-03	1.28E-05
18	WNT8B	7.71E-03	0.02709509
19	TMEM130	7.56E-03	1.37E-34
20	TM4SF20	7.35E-03	2.01E-13
21	ALDH3A1	7.15E-03	2.61E-09
22	OSTN	7.09E-03	0.022417142
23	LINC01133	6.94E-03	5.54E-56
24	LGR5	6.49E-03	2.57E-10
25	NPR3	6.17E-03	2.85E-32
26	ECM2	6.05E-03	1.08E-21
27	GPR78	5.85E-03	0.016157466
28	MLC1	5.84E-03	1.06E-05
29	RAMP1	5.64E-03	1.11E-36
30	LSP1	5.41E-03	3.81E-16

	Gene	Fold change	padj
31	ASPN	5.35E-03	0.014474934
32	ADH1B	5.26E-03	2.14E-07
33	RP11-88H9.2	4.95E-03	0.019599069
34	KRT16P4	4.57E-03	0.011788615
35	SCN3A	4.46E-03	3.20E-12
36	CLCNKB	4.32E-03	0.013592169
37	RP11-138I17.1	4.19E-03	0.010508598
38	P2RX1	3.94E-03	0.015831994
39	NTRK2	3.87E-03	3.21E-19
40	CSPG4P13	3.71E-03	0.008755354
41	KRT14	3.68E-03	2.13E-71
42	RP11-757O6.1	3.06E-03	0.006325107
43	S100B	2.92E-03	0.01002531
44	SLC12A1	2.91E-03	0.006862144
45	RP11-54O7.1	2.87E-03	0.005907141
46	NGEF	2.68E-03	4.21E-08
47	TNXB	2.67E-03	2.32E-34
48	RP11-867G23.13	2.21E-03	0.003772818
49	SGCA	2.17E-03	0.003216729
50	A2M	1.91E-03	0.002466572
51	ACAN	1.88E-03	3.59E-83
52	MMP28	1.67E-03	0.003483961
53	AGT	1.44E-03	0.001393738
54	CLEC3B	1.14E-03	3.14E-17
55	SEMA5B	1.09E-03	0.00134425
56	COMP	9.33E-04	2.11E-18
57	KRT16	5.59E-04	5.28E-22
58	CMKLR1	3.80E-04	0.000108071
59	PI16	3.54E-04	7.37E-05
60	ACTC1	3.12E-04	1.33E-19

Persistent stimulation of MSCs with TNFα+IL-1β: 60 top down-regulated genes

Human MSCs were exposed to persistent TNFα+IL-1β stimulation (concentrations as in Fig. 1) or to vehicles for 14-18 days. mRNA expression was determined by transcriptome analyses performed with 3 independent biological repeats. Differentially expressed genes were determined as those having p.adj<0.05 values. The Table presents the 60 top down-regulated genes following TNFα+IL-1β stimulation, compared to treatment by vehicle.

Supplementary Table S3

	Protein	Fold change	p value
1	POSTN	584.26	0.008413
2	MMP3	449.01	0.003417
3	AKAP12	370.01	5.68E-05
4	SERPINA9	357.71	0.001463
5	PAPPA	298.43	0.000383
6	C3	243.25	0.005594
7	CCL2	179.20	0.002724
8	ICAM1	178.73	7.48E-07
9	CXCL8	164.42	0.000579
10	CXCL1	140.92	0.001972
11	STAT1	130.05	0.000498
12	SOD2	107.95	0.000442
13	SERPINB2	103.71	0.000287
14	STC2	96.12	0.000731
15	CXCL6	87.91	0.009524
16	COL7A1	80.83	0.004863
17	CCL5	79.89	0.000121
18	TFPI2	72.85	0.021876
19	AKR1B1	66.81	0.009847
20	IL6	66.38	0.000142
21	LAMC2	55.41	0.004507
22	PLOD2	54.60	0.006475
23	PSME1	51.90	0.01911
24	STC1	51.12	0.011134
25	MMP1	47.43	0.009006
26	CNN1	42.65	0.000105
27	HSPA9	42.27	0.015654
28	CSRP2	40.51	0.002324
29	TGFB2	40.05	0.004729
30	ST3GAL1	36.66	0.009742

	Protein	Fold change	p value
31	HEBP1	33.58	0.003385
32	NT5E	32.77	0.000411
33	PRDX6	29.05	0.00195
34	ESM1	28.74	0.046582
35	NRP2	28.25	0.001512
36	CCT6A	27.02	0.001061
37	NAMPT	26.98	0.008358
38	PRDX3	26.70	0.018936
39	NARS	26.56	0.004492
40	LIF	26.47	0.00664
41	PDIA4	26.26	0.03104
42	AKAP2	26.20	0.001243
43	ATP5B	25.24	0.007947
44	MCAM	25.23	0.014412
45	TWSG1	24.93	0.012569
46	PAFAH1B2	24.85	0.014905
47	GREM1	24.39	0.000833
48	CNDP2	23.60	0.000237
49	GNB2L1	23.30	0.029093
50	PFKP	22.84	0.011232
51	HYOU1	22.15	5.37E-05
52	ANXA5	22.00	0.04737
53	GSTO1	21.73	0.030157
54	LIN7C;LIN7A	21.56	9.46E-06
55	CXCL2	21.41	0.00828
56	VPS35	20.50	0.00988
57	SLIT2	20.08	0.005472
58	UCHL1	19.43	0.000692
59	CMPK1	19.35	0.038493
60	MYLK	19.25	0.002582

Persistent stimulation of MSCs with TNFα+IL-1β: 60 top up-regulated secreted proteins

Human MSCs were exposed to persistent TNFα+IL-1β stimulation (concentrations as in Fig. 1) or to vehicles for 18-19 days. The expression of secreted proteins was determined by secretome analyses performed with 3 independent biological repeats. Differentially expressed secreted proteins were determined as those having $p<0.05$ values. The Table presents the 60 top up-regulated secreted proteins following TNFα+IL-1β stimulation, compared to treatment by vehicle.

Supplementary Table 4

	Protein	Fold change	p value
1	CKB	0.33	0.00673
2	FMOD	0.32	0.02719
3	TIMP1	0.32	6.41E-05
4	SULF1	0.31	0.012103
5	CRLF1	0.31	0.038214
6	KRT7	0.30	0.028684
7	CEMIP	0.30	0.010892
8	FNDC1	0.28	0.036441
9	DCN	0.28	0.001995
10	LTBP2	0.26	0.014139
11	PLXDC2	0.24	0.040743
12	MEGF6	0.24	0.039836
13	CXCL12	0.23	0.002151
14	FST	0.21	0.036686
15	SSC5D	0.20	0.00955
16	BGN	0.20	0.004965
17	GSTP1	0.20	0.000396
18	VASN	0.19	0.002773
19	CCDC80	0.18	0.001825
20	SERPINE2	0.18	0.000269
21	SERPINF1	0.18	0.014529
22	ITGBL1	0.16	0.000906
23	CD109	0.14	0.016153
24	LUM	0.14	0.006344
25	CD248	0.14	0.000137
26	CLU	0.14	0.029361
27	LOXL4	0.14	0.005092
28	THBS2	0.14	0.000174
29	WISP2	0.13	0.018388
30	SEMA3C	0.13	1.32E-05
31	HAPLN1	0.13	0.005343
32	COL12A1	0.12	0.001276
33	CST6	0.12	0.000115
34	PCOLCE	0.12	0.033427
35	SVEP1	0.11	0.007808
36	PAMR1	0.10	0.018589
37	MFGE8	0.10	0.003699
38	CFD	0.10	0.000232
39	TNXB	0.07	0.033837
40	ZNF683	0.06	0.023894
41	OLFML2B	0.06	0.022552
42	ENPP2	0.05	0.024361
43	IGFBP2	0.05	0.006658
44	FGF7	0.05	0.003583
45	FBLN5	0.05	0.008217
46	FBLN1	0.04	0.000378
47	MASP1	0.04	0.000458
48	CRIP1	0.04	0.000157
49	SCUBE3	0.04	0.016851
50	COL14A1	0.04	0.006045
51	COL11A1	0.03	0.000149
52	ISLR	0.03	0.013719
53	EFEMP1	0.03	0.002402
54	IGFBP6	0.03	0.000873
55	PODN	0.03	0.025621
56	THBS3	0.02	0.008996
57	ELN	0.02	0.000552
58	ACAN	0.01	0.001192
59	COMP	0.01	0.001226
60	CLEC3B	0.00	0.0018

Persistent stimulation of MSCs with TNFα+IL-1β: 60 top down-regulated secreted proteins

Human MSCs were exposed to persistent TNFα+IL-1β stimulation (concentrations as in Fig. 1) or to vehicles for 18-19 days. The expression of secreted proteins was determined by secretome analyses performed with 3 independent biological repeats. Differentially expressed secreted proteins were determined as those having $p<0.05$ values. The Table presents the 60 top down-regulated secreted proteins following TNFα+IL-1β stimulation, compared to treatment by vehicle.
