# Supplementary material for: Persistent Inflammatory Stimulation Drives the Conversion of MSCs to Inflammatory CAFs That Promote Pro-Metastatic Characteristics in Breast Cancer Cells
Source: Cancers (Basel). 2021 Mar 23;13(6):1472. doi: 10.3390/cancers13061472 (PMC8004890; doi:10.3390/cancers13061472)
Supplement: Supplementary file 1 [file cancers-13-01472-s001.zip › Ben-Baruch-Supplementary Figures 1-5-Final-28.2.21.pdf]

# Figure S1

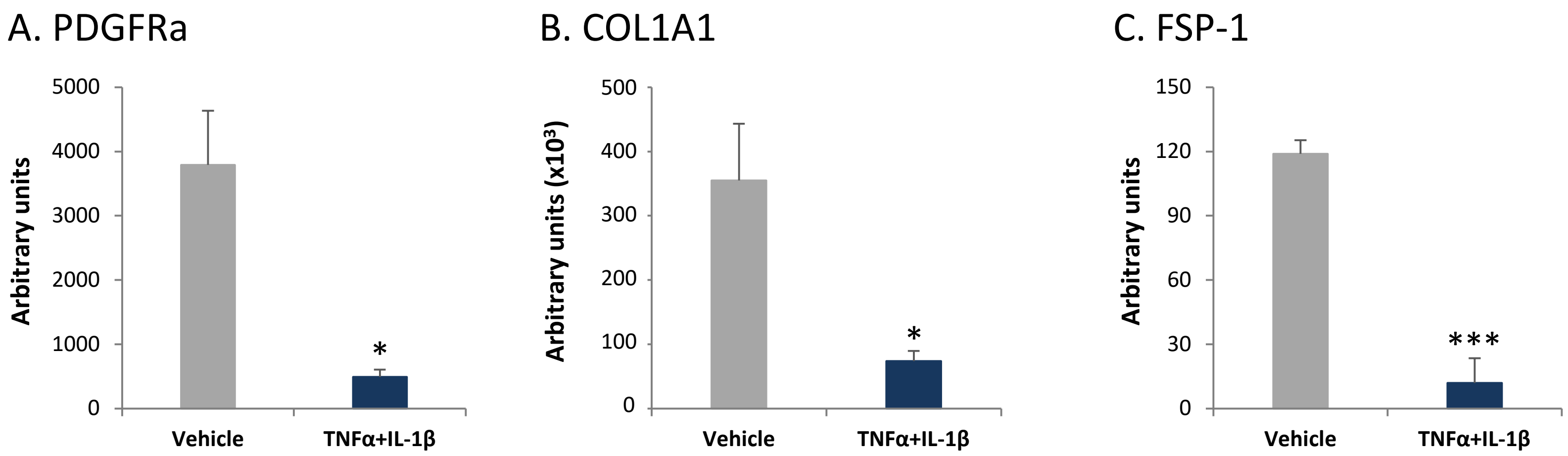


**Figure S1**

**Genes that are expressed in low levels in BM-MSC-derived CAFs, are down-regulated upon the conversion of BM-MSCs to inflammatory CAFs**

Human MSCs were exposed to persistent TNFα+IL-1β stimulation (as described in Fig. 1) or to vehicles for 14 days. Expression levels of **(A)** PDGFRa mRNA; **(B)** COL1A1 mRNA; **(C)** FSP-1 mRNA are presented. Gene expression was determined by transcriptome analyses performed with 3 biological repeats. *padj<0.05, ***padj<0.001.

# Figure S2

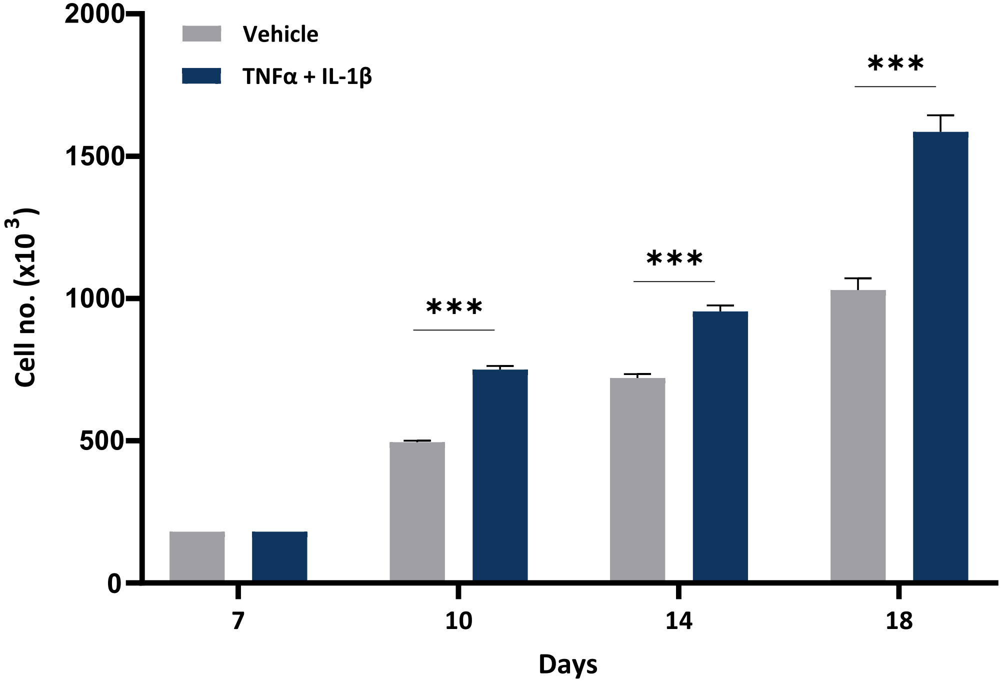


**Figure S2**

**In time-dependent analysis of MSC stimulation by TNFα+IL-1β, the stimulated cells demonstrate increased proliferation rate at all time points**

Human MSCs were stimulated by TNFα+IL-1β stimulation (as described in Fig. 1) or treated by vehicles for 7 days. Then, the cells were plated in similar numbers in the presence of the cytokines or vehicles. At days 10, 14, 18 from the start of the stimulation process (namely 3, 7 and 11 days, after culturing in equal numbers at day 7), cell numbers were determined by cell counts. ***$p<0.001$.

Figure S3

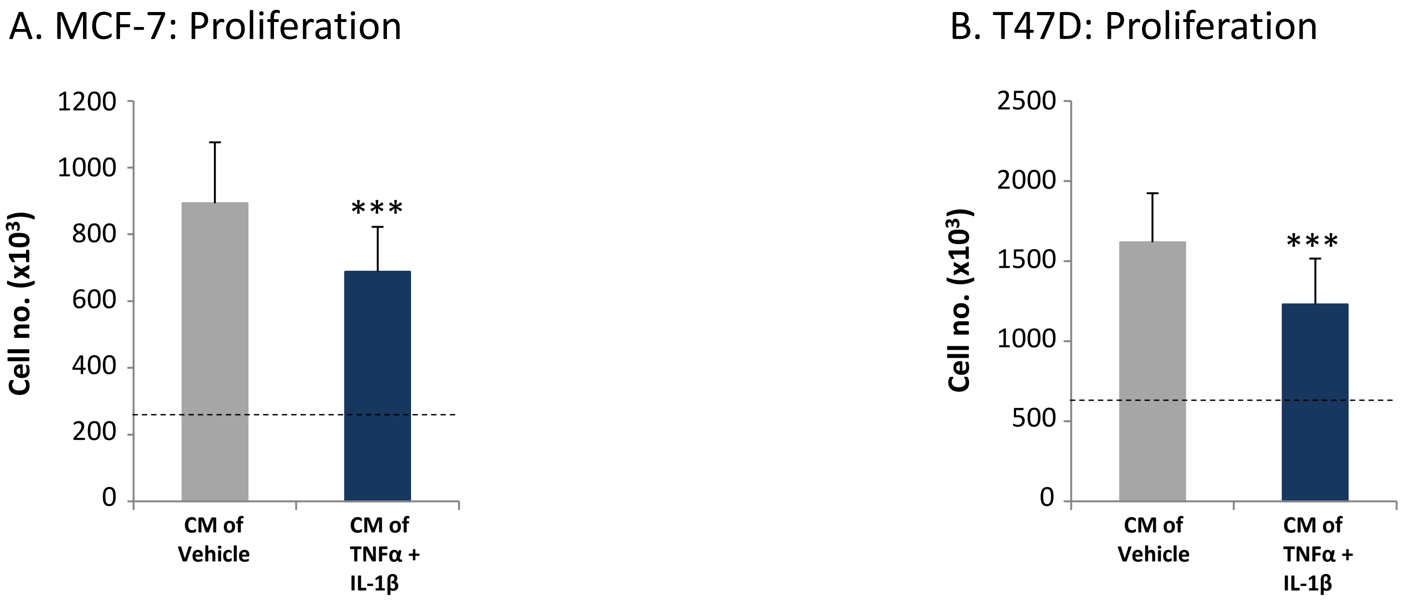


**Figure S3**

**Following persistent stimulation of MSCs with TNFα+IL-1β, the resulting inflammatory CAFs release factors that decrease the proliferation rate of luminal-A BC cells**

Human MSCs were exposed to persistent TNFα+IL-1β stimulation (as described in Fig. 1) or to vehicles for 14-18 days. Cytokine-devoid CM were collected (as described in Fig. 7) and were administered to human luminal-A BC cells (plated in similar numbers before the addition of CM derived from each of the two MSC types) for 40 hrs. Dashed line represents the number of tumor cells plated at the beginning of the experiment, before the addition of CM: **(A)** MCF-7: 260x$10^3$ cells. **(B)** T47D: 630x$10^3$ cells. Cell numbers were determined by cell counts, at the end of the CM-stimulation process. ***p<0.001. Average ± SD of 4 independent experiments is presented.

# Figure S4

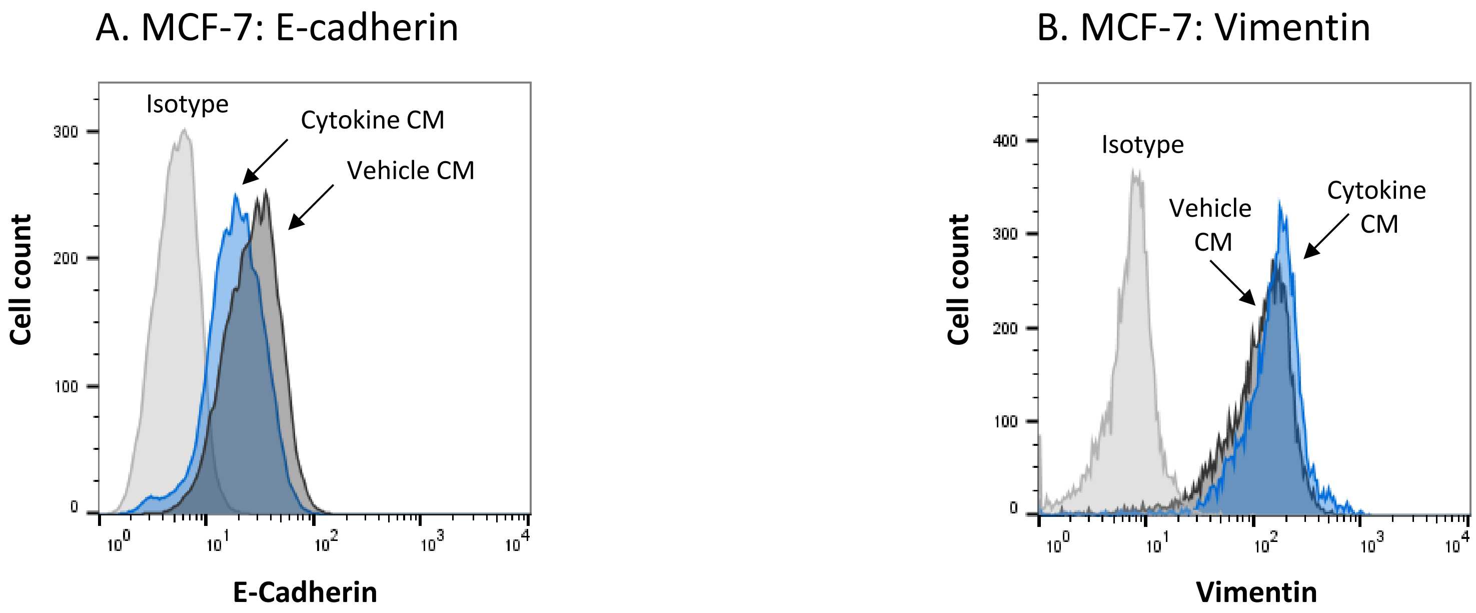


**Figure S4**

**Following persistent stimulation of MSCs with TNFα+IL-1β, the resulting inflammatory CAFs release factors that induce a partial EMT phenotype in luminal-A BC cells**

Human MSCs were exposed to persistent TNFα+IL-1β stimulation (as described in Fig. 1) or to vehicles for 14-18 days. Cytokine-devoid CM (termed herein "Cytokine CM") were collected (as described in Fig. 7) and were administered to MCF-7 luminal-A BC cells in parallel to CM of control vehicle-treated MSCs (termed herein "Vehicle CM") for 72 hrs. The extracellular expression of E-cadherin and the intracellular expression of vimentin were determined by flow cytometry. Images from a representative experiment out of n=3 (of one donor) are presented.

# Figure S5

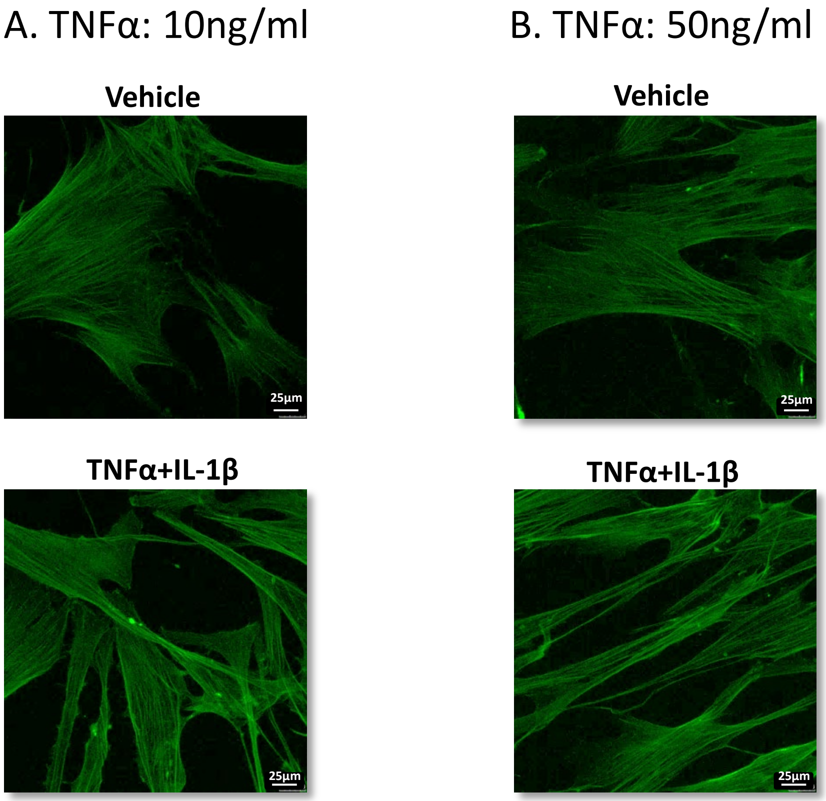


**Figure S5**
**Titration of TNFα concentration required for induction of CAF-like morphology in persistently-stimulated MSCs**
Human MSCs were exposed to persistent TNFα+IL-1β stimulation (as described in Fig. 1) or to vehicles for 18 days. Then, cell morphology was determined by confocal analysis, using phalloidin to stain for actin filaments. Bar, 25µm. **(A)** Stimulation by 10ng/ml TNFα + 0.5ng/ml IL-1β. **(B)** Stimulation by 50ng/ml TNFα + 0.5ng/ml IL-1β.
